# An Optimized Competitive-Aging Method Reveals Gene-Drug Interactions Underlying the Chronological Lifespan of *Saccharomyces cerevisiae*

**DOI:** 10.3389/fgene.2020.00468

**Published:** 2020-05-14

**Authors:** J. Abraham Avelar-Rivas, Michelle Munguía-Figueroa, Alejandro Juárez-Reyes, Erika Garay, Sergio E. Campos, Noam Shoresh, Alexander DeLuna

**Affiliations:** ^1^Unidad de Genómica Avanzada (Langebio), Centro de Investigación y de Estudios Avanzados del IPN, Irapuato, Mexico; ^2^Broad Institute of MIT and Harvard, Cambridge, MA, United States

**Keywords:** post-mitotic aging, genetic analysis, high-throughput screening, gene-drug interactions, *Saccharomyces cerevisiae*

## Abstract

The chronological lifespan of budding yeast is a model of aging and age-related diseases. This paradigm has recently allowed genome-wide screening of genetic factors underlying post-mitotic viability in a simple unicellular system, which underscores its potential to provide a comprehensive view of the aging process. However, results from different large-scale studies show little overlap and typically lack quantitative resolution to derive interactions among different aging factors. We previously introduced a sensitive, parallelizable approach to measure the chronological-lifespan effects of gene deletions based on the competitive aging of fluorescence-labeled strains. Here, we present a thorough description of the method, including an improved multiple-regression model to estimate the association between death rates and fluorescent signals, which accounts for possible differences in growth rate and experimental batch effects. We illustrate the experimental procedure—from data acquisition to calculation of relative survivorship—for ten deletion strains with known lifespan phenotypes, which is achieved with high technical replicability. We apply our method to screen for gene-drug interactions in an array of yeast deletion strains, which reveals a functional link between protein glycosylation and lifespan extension by metformin. Competitive-aging screening coupled to multiple-regression modeling provides a powerful, straight-forward way to identify aging factors in yeast and their interactions with pharmacological interventions.

## Introduction

A major challenge in aging research is to describe the way in which different genetic pathways and biochemical processes mediating aging are interconnected to one another (Kirkwood, 2008; López-Otín et al., 2013). Simple cellular models provide a starting point to grant a systems-level understanding of aging, in which the lifespan phenotype is addressed as a complex trait resulting from the action of multiple genes, cellular processes, environmental factors, and their interactions.

The chronological lifespan (CLS) of *Saccharomyces cerevisiae* is used to describe genetic, nutrimental, and pharmacological factors underlying survivorship of post-mitotic, non-dividing cells (Longo and Fabrizio, 2012). The budding yeast’s replicative-lifespan and CLS are simple experimental models that have been used to reveal the conserved lifespan-extending effects of reduced TOR and RAS/PKA signaling, as well as the anti-aging effect of rapamycin, spermidine, and caloric restriction (Wei et al., 2008; Eisenberg et al., 2009; Longo and Fabrizio, 2012; Gems and Partridge, 2013). Traditionally, the CLS of a yeast-cell population is measured by counting colony-forming units from samples of a long-term stationary-phase culture (Longo et al., 2012; Hu et al., 2013). More recently, large-scale screening approaches have been implemented to screen for genetic aging factors in yeast. These studies provide unbiased catalogs of CLS mutant phenotypes (Powers et al., 2006; Fabrizio et al., 2010; Matecic et al., 2010; Burtner et al., 2011; Garay et al., 2014), mutants with diminished or enhanced response to dietary restriction or nutrient limitation (Gresham et al., 2011; Campos et al., 2018), and CLS phenotypes of collections of wild isolates and lines derived from biparental crosses (Jung et al., 2018; Barre et al., 2020).

A current limitation in the field is that large-scale CLS-phenotyping screens have resulted in a large number of false positive hits when further confirmed by smaller-scale approaches, ranging from 50 to 94% (Powers et al., 2006; Fabrizio et al., 2010; Matecic et al., 2010; Burtner et al., 2011; Garay et al., 2014). In addition, comparisons of different large-scale studies show that there is little overlap among the identified genetic factors, which could be explained in part by differences in genotypic background, media composition, and subtle environmental variations (Smith et al., 2016). In addition, changes in controlled or uncontrolled environmental conditions are known to be important modifiers of CLS phenotypes and confounding causes of aging (Burtner et al., 2009, 2011; Santos et al., 2015; Smith et al., 2016; Campos and DeLuna, 2019). In this context, a combination of high throughput and resolution is much needed to correctly determine not only genetic aging factors, but also to quantitatively derive their interactions with nutrimental, chemical, or pharmacological environments.

In an effort to improve the throughput of CLS screening without sacrificing phenotyping sensitivity, we previously introduced a competition-based method for quantitative large-scale genetic analysis that simultaneously measures an internal reference with each gene-deletion strain (Garay et al., 2014). In brief, each RFP-labeled single-deletion strain is mixed with a CFP wild-type reference and grown to saturation; fluorescence signal in outgrowth cultures is used to estimate the relative number of viable cells in the non-dividing culture at different time points in stationary phase. One of the main advantages of such competition-based assay is the use of an internal reference strain, whereby mutant and wild-type strains age under the same conditions, allowing direct quantification of their relative survivorship. This approach recapitulates known CLS factors and suggests new lifespan phenotypes in yeast (Garay et al., 2014). More recently, we have used this strategy to screen for dietary restriction factors, namely CLS gene-deletion phenotypes that are aggravated or alleviated when yeast populations are aged under a poor nitrogen source (Campos et al., 2018).

In this study, we describe an optimized multiple regression modeling strategy to analyze measurements from our competition-based approach for CLS genetic analysis in yeast, by accounting for possible differences in growth rate and experimental batch effects. In addition, we provide a systematic analysis of the method’s replicability and data-analysis scripts. For ten knockout strains, we compare the replicability of our results with those obtained with a useful parallelizable approach based on outgrowth kinetics (Murakami et al., 2008; Jung et al., 2015). Importantly, we take advantage of our improved data-analysis method to derive gene-drug interactions by measuring the relative effects on survival of metformin in 76 deletion strains of widely conserved genes. We discuss the potential of competitive-aging screening to describe large numbers of genetic and environmental interactions underlying aging and longevity in aging cells.

## Materials and Methods

### Strains and Media

Ten single-gene deletion strains targeting *ATG1*, *HAP3*, *MSN2*, *MSN4*, *RAS2*, *RIM15*, *RPS16A*, *STE12*, *GLN3*, and *SWR1* were generated *de novo* by PCR-based gene replacement in the YEG01-RFP background (Garay et al., 2014) using the *natMX4* module from pAG25 (Euroscarf). In addition, two isogenic reference strains were generated over the YEG01-RFP and YEG01-CFP backgrounds by deleting the neutral *HO* locus. Lithium-acetate transformation and 100 μg/ml clonNAT (Werner BioAgents) were used. The resulting strains were *MAT-*α *x*Δ*:natMX4 PDC1-RFP.CaURA3MX4 can1*Δ*:STE2pr-SpHIS5 lyp1*Δ *ura3*Δ*0 his3*Δ*1 LEU2 MET15*; strong constitutive expression of fluorescent proteins during exponential growth is achieved by carboxyl-terminal fusion to the Pdc1 protein.

For gene-drug interactions, a collection of RFP-tagged gene-deletion strains was generated by mating an array of 85 strains from the yeast deletion collection to the *ho*Δ YEG01-RFP SGA-starter strain, as previously described (Garay et al., 2014). Resulting prototrophic strains were *MAT-*a *x*Δ*:kanMX4 PDC1-RFP.CaURA3MX4 can1*Δ*:STE2pr-SpHIS5 lyp1*Δ *ura3*Δ*0 his3*Δ*1 LEU2 MET15*. The CFP reference strain was the neutral marker reference *his3*Δ:*kanMX4*. Double-marker strains and CLS data were successfully obtained for 76 deletion strains (Supplementary Data S1).

Aging medium was synthetic complete (SC) with ammonium, 2% glucose, and 0.2% amino acid supplement mix (Amberg, 2005), without buffering. 40 mM metformin (Sigma D150959) was used where indicated. Single-culture and competitive outgrowth kinetics were done in low fluorescence YNB medium (YNB-lf) with 2% glucose and complete amino-acids supplement mix (Sigma Y1501 completed with Sigma U0750) (Sheff and Thorn, 2004). All cultures were incubated in a growth chamber at 30°C and 80–90% relative humidity without shaking; aging co-cultures were vigorously shaken at every sampling point. For media recipes, see Supplementary Table S1.

### Competitive-Aging Culture Setup and Outgrowth Measurements

To obtain relative CLS measurements, mutant (*x*Δ_*RFP*_) and reference strains (WT_*RFP*_ and WT_*CFP*_) were pre-cultured separately until saturation. Saturated cultures were mixed in a 2:1 mutant:reference ratio and ∼1.5 μL aliquots were transferred with a 96 solid-pin replicator (V&P Scientific VP 407) onto 96 semi deepwell plates (Nunc 260251) with 750 μL of fresh aging medium and disposable plastic covers.

Outgrowth sampling of the competitive-aging culture began 3 days after initial inoculation (time zero) and was repeated initially every 24 h and 48–72 h afterward for up to 16 days after time zero. At each sampling day, *T*_*i*_, 12 μL of shaken aging cultures were inoculated onto 140 μL fresh YNB-lf medium in clear polystyrene 96-well plates (Corning 3585). Outgrowth was monitored every 1–3 h until cultures reached stationary phase, by measuring raw fluoresence of mCherry (RFP, Ex 587/5 nm and Em 610/5 nm), Cerulean (CFP, Ex 433/5 nm and Em 475/5 nm), and absorbance at 600 nm (*OD*_600_) using a Tecan Infinite M1000 reader integrated to a robotic platform (Tecan Freedom EVO200). Outgrowth cultures were resuspended by vigorous shaking right before every measurement. To increase fluorescence-signal dynamic range, an outgrowth culture with the samples was measured 1 day before the actual experiment to calculate the optimal-gain at late exponential-growth, when fluorescence signal is at its maximum level. Optimal gain values were fixed for all measurements in the experiment; values used were 140–167 and 131–137 for RFP and CFP, respectively.

### Death-Rate Calculation From Competitive-Aging Outgrowth Data

The $\frac{RFP}{CFP}$ signal ratio was used to estimate the number of cells expressing each fluorescent signal. Auto fluorescence background was defined as the RFP and CFP signal of WT_*CFP*_ and WT_*RFP*_ monocultures, respectively, and was subtracted from all competitive aging cultures. Using data from different measurements in the outgrowth cultures (*t*_*j*_, in hours) of each stationary-phase sampling point (*T*_*i*_, in days), the signal ratio $ln{\left(\frac{RFP}{CFP}\right)}_{T_{i},t_{j}}$ of each sample (*w*) was fitted to the linear model *A*_*w*_ + *S*_*w*_⋅*T*_*i*_ + *G*_*w*_⋅*t*_*j*_ + *C*_*T*_*i*_,*t*_*j*__ The ratio of viable cells at the beginning of the experiment is modeled in *A*, while *S* (relative survivorship) is the death-rate difference of the mutant and wild-type reference, and *G* (relative growth) is their growth-rate difference in the outgrowth co-culture. In addition, the term *C*_*T_i_, t_j_*_ was introduced to consider the systematic variation of each plate at each stationary-phase sampling point *T*_*i*_*t*_*j*_. A complete description of the model and its implementation is provided in Supplementary Note S1.

### Deriving Gene-Drug Interactions From CLS Phenotypes

To identify gene-drug interactions, namely cases in which the CLS phenotype of a gene-deletion strain is significantly aggravated or alleviated by treatment with a drug, the relative survivorship of a set of 76 deletion strains was measured with and without 40 mM metformin. Deletion strains were randomly selected from the yeast deletion collection, considering only genes with a mammalian ortholog (Ensembl). Relative survivorship values were rescaled to a dimensionless parameter, given that metformin increases yeast CLS and that the relative survivorship *S* expressed in days is constrained by the death rate of the wild-type reference, *r*_*wt*_, which is different in each condition. For each mutant under a given condition, rescaled survivorship (*rS* was defined as $rS=-\ln\left(1-\frac{S}{r_{wt}}\right)$, where *r*_*wt*_ is the average death rate of all control wild-type competitions in each plate. Gene-drug interactions were defined as cases in which a mutant’s *rS* in SC was significantly different to the *rS* in SC + metformin (*p* < 0.05; *t*-test).

### Measuring Death Rate in Monoculture From Outgrowth Kinetics

Chronological lifespan estimates based on outgrowth kinetics of single-population aging cultures were adapted from a well established high-throughput method (Murakami et al., 2008; Jung et al., 2015). Culture density was monitored by measuring absorbance at *OD*_600*nm*_; background signal—*OD*_600_ at outgrowth inoculation—was subtracted to each data point. At each aging sampling point *T*_*i*_ (days), the time-shift in hours, *t*, to reach a fixed cell density of *OD*_600_ = 0.35 reports for the remaining fraction of viable cells in the population, as previously reported (Murakami et al., 2008). Specifically, for each successive age time point, the percent of viability (*V*_*T*_) was calculated using the equation $V_{T}=\frac{1}{2\left(\Delta t_{n}/\delta \right)}$, where Δt_*n*_ is the time shift and δ is the doubling time. Death rates, *r*_*i*_, were the exponential decay rates calculated by fitting all *V*_*T*_ data points as a function of time in stationary phase to a first-order exponential model (Matlab, *fit*). We have previously verified that an exponential, rather than linear decay model, better explains the loss of viability of wild-type cells aged under different nitrogen sources (Campos et al., 2018). Half-life was calculated as $HL_{i}=\frac{\text{ln}\left(2\right)}{r_{i}}$.

### Live/Dead Cell Viability Assays

Stationary-phase cultures (5 μL) were transferred to 50 μL of BD FACSFlow (BD #342003) with 0.1% propidium iodide (PI) and 0.1% SYTO9 green (ThermoFisher L34952) in 96-well plates (Corning 3585), shaken for 1 min at 1,000 rpm in a plate shaker (Heidolph Titramax 1000), and incubated at room temperature in the dark for 20 min. An analytical flow cytometer with a high-throughput sampler (LSRFortessa-HTS, Becton Dickinson) was used to capture 10,000 events. SYTO9 fluorescence was excited with a 488 nm blue laser and collected through a 525/50 nm band-pass filter and a 505LP emission filter, while PI signal was excited with a 488 nm blue laser and collected through a 712/21 band-pass filter. Events with a increased SYTO9/PI signal were counted as alive.

## Results

### Relative Survivorship in Stationary Phase Can Be Estimated From Bulk Fluorescence Signal of Two Populations in Co-culture

**FIGURE 1:**
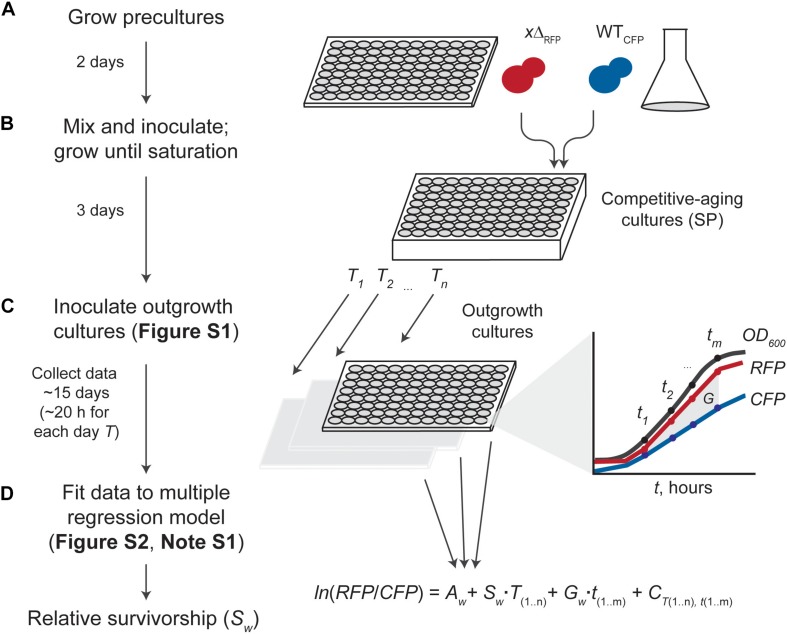
Experimental and data-analysis workflow. **(A)** Starting cultures of fluorescence-tagged gene deletion (*x*Δ_*RFP*_) and wild-type reference (WT_*CFP*_) are grown separately. **(B)** Saturated cultures are mixed (usually in a 1:2 WT/*x*Δ ratio for increased dynamic range) and inoculated into SC aging medium in a semi-deep well plate until stationary phase (SP). **(C)** Competitive-aging cultures are sampled regularly at days *t*; outgrowth cultures in fresh medium are monitored at *T* hours with simultaneous measurement of absorbance at 600 nm (*OD*_600_), and raw RFP and CFP signals (Supplementary Figure S1). Possible differences in growth rate (*G*) are taken into account. **(D)** Data analysis uses the change in fluorescent-signal ratio in the outgrowth cultures to estimate the relative survivorship of each mutant (*S*_*w*_). Data is fitted to a multiple linear-regression model considering the change of ln(RFP/CFP) empirical measurements over time as a function of the starting strains’ proportion (*A*), the mutant’s relative growth rate (*G*), systematic batch errors in the measurements (*C*), and the mutant’s survivorship relative to the wild-type (*G*), our parameter of interest (Supplementary Figure S2 and Note S1).

We sought to optimize data analysis and systematically test a competition-based method aimed at describing CLS phenotypes of viable gene-deletion strains in budding yeast. To directly measure the lifespan effects in a high-throughput manner, we co-cultured RFP- and CFP-tagged deletion (*x*Δ_*RFP*_) and wild-type (WT_*CFP*_) strains, respectively, in 96 parallel cultures (Figure 1). We tracked changes in the relative abundances of deletion wild-type in each co-culture, as a function of time in stationary phase (*T*, days). To this end, we inoculated stationary-phase cells at different time points into fresh medium and monitored the outgrowth at multiple times (*t*, hours) by measuring absorbance at 600 nm (*OD*_600_), bulk RFP signal (RFP), and bulk CFP signal (CFP), until the outgrowth co-cultures reached saturation.

We characterized the CLS of ten deletion strains impaired in genes from processes that are known to regulate lifespan. By focusing on this specific set of genes, we were able to carefully assess the performance of our method on a wide range of effects, from strong to mild short- and long-lived phenotypes. For instance *rim15*Δ (Wei et al., 2008; Burtner et al., 2011), *ste12*Δ (Campos et al., 2018), *hap3*Δ (Laschober et al., 2010), and *atg1*Δ (Alvers et al., 2009) are all known to decrease lifespan at different quantitative levels in yeast. In contrast, long-lived CLS phenotypes have been reported for *swr1*Δ (Garay et al., 2014), *ras2*Δ (Fabrizio and Longo, 2003; Wei et al., 2008), and *gln3*Δ (Powers et al., 2006). Deletion of *MSN2* or *MSN4* either has been shown to have either no effect or to decrease lifespan (Fabrizio et al., 2001; Wei et al., 2008; Schroeder et al., 2013; Campos et al., 2018). While inactivation of ribosomal genes results in different CLS phenotypes (Burtner et al., 2011; Garay et al., 2014), to our knowledge there is no specific information for *rps16a*Δ deletion. Each *x*Δ_*RFP*_ was co-cultured until saturation with the WT_*CFP*_ reference in up to seven replicates in a single deep-well plate (see section “Materials and Methods”). Competitive-aging cultures were monitored for ∼15 days in stationary phase. As expected, outgrowth kinetics measured by *OD*_600_ showed a clear shift with time (days) in stationary phase; aging co-cultures gradually took a longer time in outgrowth (hours) to reach a given cell density (Supplementary Figure S1). This prevalent shift in growth kinetics reflects the loss of viability with culture age, as previously described (Murakami et al., 2008).

In terms of fluorescence-signal kinetics, we observed that the WT_*RFP*_ or WT_*CFP*_ monocultures mostly recapitulated *OD*_600_ kinetics, suggesting that loss of viability can also be measured by the shift of the fluorescence signal over the days (Supplementary Figure S1, red and blue lines). As expected, WT_*RFP*_ + WT_*CFP*_ populations competed in co-culture showed similar shifts in fluorescence signals, suggesting that loss of viability occurred at similar rates in both wild-type populations (gray lines). In contrast, some *x*Δ_*RFP*_ + WT_*CFP*_ co-cultures showed exacerbated delayed or accelerated shifts in fluorescence kinetics. For instance, the *hap3*Δ_*RFP*_ + WT_*CFP*_ co-culture showed a steep decrease in bulk RFP signal as a function of days in stationary phase, along with a slight increase in CFP signal (orange lines), suggesting that loss of viability occurred at a rate faster than the WT_*CFP*_ reference (short-lifespan phenotype). In contrast, the *swr1*Δ_*RFP*_ + WT_*CFP*_ co-culture showed a small increase in RFP signal along with steady CFP signal (green lines), suggesting slower loss of viability of *swr1*Δ compared to the WT (long-lifespan phenotype). We note that CFP- and RFP-signal dynamics at any given outgrowth are not necessarily the same, mainly because of differences in signal behavior and dynamic ranges. Given that data is collected throughout the outgrowth culture, and that a universal CFP-labeled wild-type reference is used, these systematic differences are readily adjusted during data analysis (see below). In summary, raw-data examples illustrate that changes in bulk fluorescence in co-culture can be used to estimate loss of cell population viability as a function of time in stationary phase.

### A Model to Adjust Relative Survivorship in Co-culture From Changes in Relative Fluorescence Signal

To obtain a quantitative phenotypic value from our experimental measurements, we developed a model that provides a relative survivorship parameter, *S*. Specifically, we established a multiple linear regression analysis where each experimental measurement in the outgrowth culture is modeled as:

$$\text{ln}{\left(\frac{\text{RFP}}{\text{CFP}}\right)}_{T_{i},t_{j}}=A_{w}+S_{w}\cdot T_{i}+G_{w}\cdot t_{j}+C_{T_{i},t_{j}}$$

Using a system of linear equations, we obtained the regression coefficients using all outgrowth measurements in a 96-well plate. The contribution of relative survivorship (*S*) along with that of the other three parameters for competing populations are illustrated in Supplementary Figure S2, with selected examples. The expected value, $\text{ln}{\left(\frac{\text{RFP}}{\text{CFP}}\right)}_{T_{i},t_{j}}$, is the logarithmic quotient of the sizes of the populations of *x*Δ_*RFP*_ and WT_*CFP*_ from an outgrowth inoculated at day *T*_*i*_ in stationary phase and measured after *t*_*j*_ hours in the outgrowth culture. *S*_*w*_ is the difference in death rates, which are modeled with exponential decay (Campos et al., 2018), while *G*_*w*_ is the difference in growth rates (open circles and crosses, for *S* and *G*, respectively). Parameter *A*_*w*_ is the logarithmic proportion of the sizes of both populations at the beginning of the experiment (solid vertical lines at *T*_1_ = 0). Finally, the term *C*_*T_i_, t_j_*_ is the error in each measurement, which is mostly determined by the deviation from zero change in WT_*RFP*_ + WT_*CFP*_ reference competitions, and is similar to the deviation of measurements in *x*Δ_*RFP*_ + WT_*CFP*_ competitions (Supplementary Figure S2, panel B). The rationale here is that relative survivorship (*S*) and relative growth rate (*G*) are by definition equal to zero in reference competitions (WT_*RFP*_ + WT_*CFP*_), and therefore we assume that consistent changes in $\text{ln}\left(\frac{{\text{RFP}}_{\text{ref}}}{{\text{CFP}}_{\text{ref}}}\right)$ are due to systematic technical errors in the measurements. It must be noted that there are usually survivorship and growth-rate differences between the two wild-type strains (WT_*RFP*_ and WT_*CFP*_), which are taken into account while fitting the data. For instance, in this experiment the WT_*RFP*_ (and isogenic deletion strains) died faster than the WT_*CFP*_ reference used, which was evident both from the raw data of WT-reference competitions (Supplementary Figure S1) as well as the negative slope of WT_*RFP*_ + WT_*CFP*_ data points in Supplementary Figure S2. This phenotypic difference is normalized by fitting data to the model in which the WT has a fixed *S* = 0 value.

Model development and implementation is further described in Supplementary Note S1. In the following sections, we show that competitive-aging experiments with multiple-regression modeling provide reliable and replicable quantifications of relative survivorship. We also show that this procedure is useful to identify CLS phenotypes and to score their interactions with pharmacological factors.

### Competitive-Aging Experiments Provide Replicable Estimates of Survivorship

**FIGURE 2:**
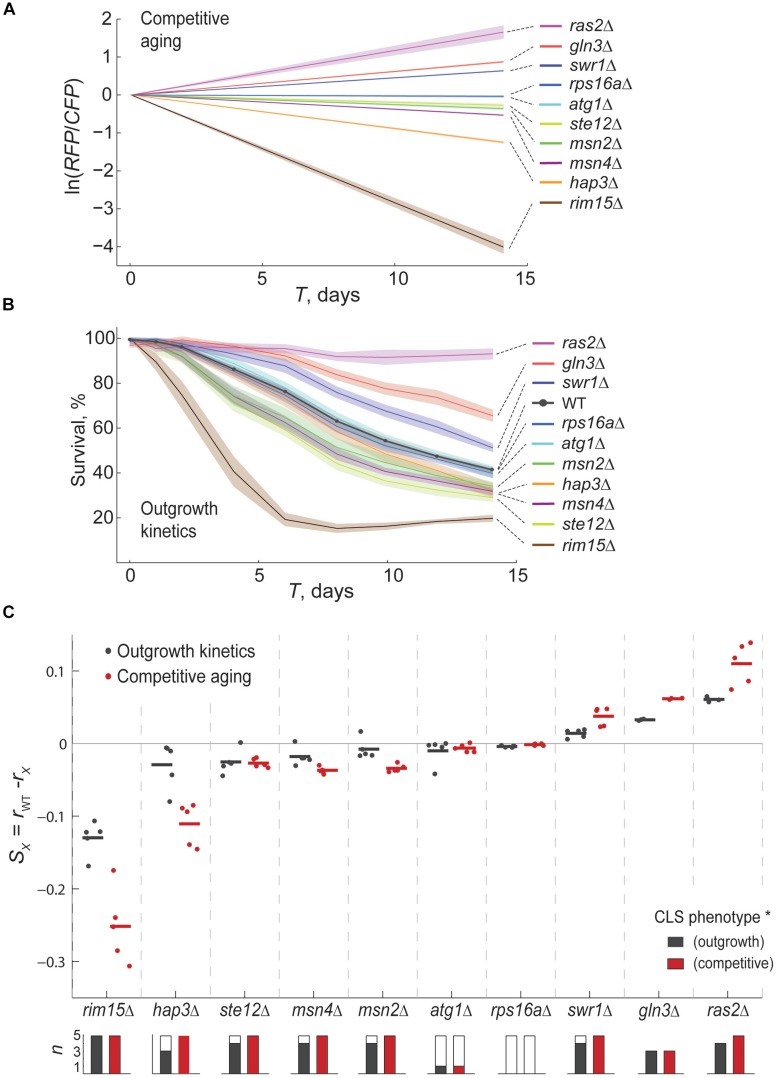
The competitive-aging method is accurate and replicable. **(A)** Relative survivorship estimated by competitive aging. The modeled ln(RFP/CFP) is shown for the WT and ten mutant strains at each *T*_*i*_ point, averaged for at least six technical replicates along with the CI 95% (shaded area). **(B)** Percent of surviving cells in monoculture over time measured by the shift in outgrowth kinetics; the mean and CI 95% are shown. **(C)** Average relative survivorship of three to five experimental replicates is shown, expressed either as the difference of individual death rates for monoculture outgrowth kinetics (*r*_*wt*_−*r*_*x*_, black) or as survivorship coefficients for competitive aging (*S*, red). Each data point is the average of at least six technical replicates of monocultures or competitions in an independent experimental replicate. Horizontal lines are the mean of each data series. Stacked bars below the plot indicate the number of replicate plates per sample; solid fraction indicates the number of significant samples compared with the WT distribution (*p* < 0.01, *t*-test; each experimental batch has 31 reference and at least six deletion samples).

To systematically assess the technical replicability of the competitive-aging method, we measured the CLS of ten mutants and reference strains in 96-well plates with multiple independent replicate wells. Specifically, we measured relative survivorship of up to seven replicate samples of each one of the ten deletion mutants together with 31 wild-type reference competitions in a 96-well plate (Figure 2A); the entire experiment was carried out twice: one experiment with three replicate plates and the second one with two replicate plates. We validated our results with another large-scale method that provides precise estimates of survivorship (Figure 2B). In particular, we measured CLS of the same array of mutants and wild-type strains using an established high-throughput method that is based in the changes of outgrowth kinetics of aging monocultures (Murakami et al., 2008; Jung et al., 2015). Qualitative inspection showed that both methods correctly scored those mutants with known CLS effect (Fabrizio et al., 2001; Fabrizio and Longo, 2003). Specifically *rim15*Δ and *hap3*Δ had reduced survivorship, while *ras2*Δ and *gln3*Δ showed increased lifespan compared to wild type.

To quantitatively contrast the replicability of both experimental approaches, we fitted decay curves from the outgrowth kinetics experiments to an exponential model. We then compared the difference of adjusted exponential death rates of wild-type and mutant strains from monoculture aging to the *S* parameter obtained from competitive-aging. We observed that both methods performed similarly when comparing the technical variation within each of the plate replicates; there was no significant difference in the typical standard error of the mean in outgrowth kinetics and competitive aging (Supplementary Figure S3). Likewise, when looking at the correlation of quantitative data resulting from independent replicates, we found equivalent correlation coefficients between replicates of each of the two approaches, showing that competitive-aging is as replicable as the outgrowth-kinetics method (Supplementary Figure S3). We note that *gln3*Δ was not included in one of the experiments, and that the *ras2*Δ was atypically noisy in one of the outgrowth-kinetics replicates (not shown); hence these strains were conservatively excluded from this analysis to prevent overestimation of the intra- and inter-batch variability of the outgrowth-kinetics approach. Together, these results indicate that the competition-based method is at least as replicable as the method we compared it to, despite inherent variation of different experimental batches.

We looked closer into the distribution of effects of all deletion mutants in five replicates, as determined by the two experimental approaches (Figure 2C). Competitive-aging screening showed higher dynamic range but also higher variability. Deletion of *rim15*Δ consistently showed strong short-lived phenotypes with both approaches, while milder short-lived phenotypes of *hap3*Δ, *ste12*Δ, *msn4*Δ, and *msn2*Δ were also scored in most of the plate replicates. Virtually all long-lived phenotypes were correctly scored by both approaches, except for one replicate of *swr1*Δ by the OD-kinetics method. The short- and long-lived phenotypes of *atg1*Δ and *rps16a*Δ, respectively, were not recapitulated by any of the two methods, most likely due to differences in our experimental conditions or strain background.

Having multiple reference samples (WT_*RFP*_ + WT_*CFP*_) in each 96-well plate improves the fit of the model, but necessarily reduces the number of samples in large-scale analyses, which usually require running many batches in parallel. To maximize experimental throughput without losing quantitative resolution, we used this experiment to estimate the optimal number of reference samples per plate to estimate of *S*. To do so, we quantified how the number of WT-reference samples (from 1 to 31 in this experiment) used to fit *S* = 0 affected the variation of mutant’s *S*. We observed that both the average standard deviation of *S* and the confidence intervals of the fit of *S* showed a steep decline from one to five reference samples, after which variation kept decreasing with diminishing returns (Supplementary Figure S4). Thus, we conclude that including 6 to 10 reference samples is enough to provide a robust description of relative-CLS phenotypes in large-scale genetic analyses of mutants, environments, and their interactions.

Finally, we used this data set to evaluate the influence of the growth rate of the mutants in the context of our competitive-aging method and multiple-regression model, which includes parameter *G*. Importantly, we observed that the model correctly identified mutants with growth defects, as measured by independent culture outgrowths (Supplementary Figure S5); hence, this parameter may enhance a more accurate quantification of relative survivorship of deletion strains. To compare the estimation of the effects on survivorship upon introduction of the *G* parameter, the data was fitted assuming *G* = 0 in all mutant-wild-type competitions; we observed that the difference in the estimation of *S* when *G* was included was modest, but mostly explainable by *G*. As expected, the relative survivorship of slow-growth mutants (*rim15*Δ, *gln3*Δ, and *ras2*Δ) was underestimated when differences in growth rate were not taken into account (Supplementary Figure S5). These results indicate that multiple-regression model optimizes data analysis, especially in screens including mutants with impaired exponential growth.

### Competitive-Quantification of CLS Under Different Conditions Successfully Describes Gene-Drug Interactions

Lifespan is a complex trait determined by different cellular pathways, hundreds of genes, and environmental variables (López-Otín et al., 2013; Campisi et al., 2019). A current challenge in the field is to understand how different factors are integrated with one another to control cell survivorship. Our competitive-aging method provides high-resolution and replicable data, which enables an accurate quantitative description of CLS-phenotype interactions. As a proof of principle experiment using our optimized experimental design and data analysis, we screened for gene-environment interactions in an array of knockout mutants aged with and without the lifespan-extending drug metformin.

**FIGURE 3:**
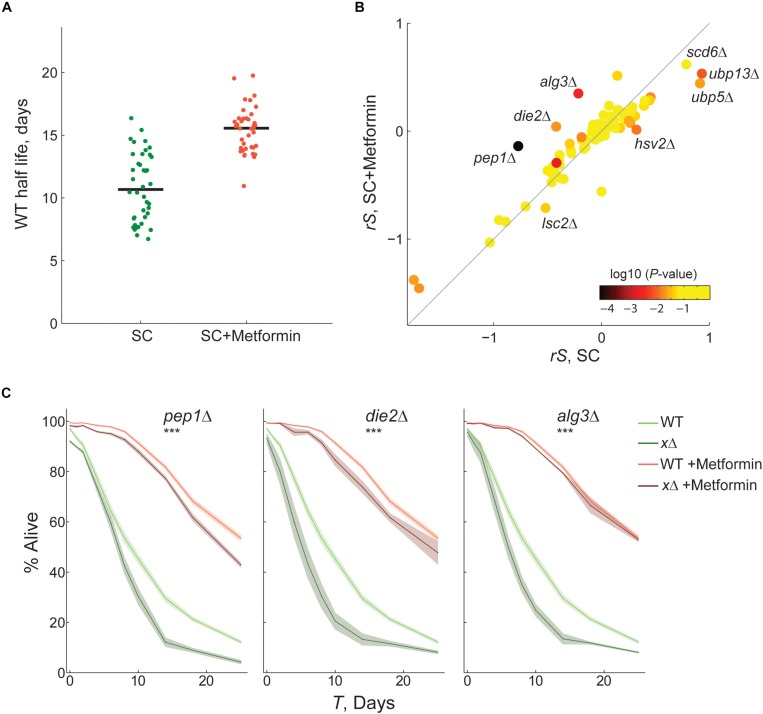
Identification of gene-drug interactions by competitive-aging screening. **(A)** Half life of WT-reference samples with (*n* = 39) or without metformin (*n* = 40). **(B)** Scatter plot comparing the CLS phenotypes of 76 gene-deletion strains with or without metformin; the average rescaled relative survivorship *rS* shown of four replicates is shown. Color scale indicates the *p-*value of paired *t*-tests between the *S* in SC and SC + Metformin. **(C)** Gene-drug interactions confirmed by live/dead staining. Survival curves of wild-type (light colors) and gene-deletion (dark colors) strains in nominal SC medium (green) or SC + metformin (orange); 95% CI were calculated from at least three replicates (shaded area). The interaction between metformin and gene-deletions was scored significant by two-way ANOVA tests of the death rates (***p < 0.01).

We confirmed that the CLS of WT reference samples of yeast increased significantly from a half life of 10.7–15.5 days when treated with 40 mM metformin (Figure 3A; *p* < 10^–9^, Wilcoxon rank sum test). Cultures aged in SC medium with metformin showed decreased acidification (Supplementary Figure S6). This suggests that modulation of extra-cellular pH is among the modes of action of the drug resulting in increased survivorship, given that acetic-acid induced mortality is a mechanism of chronological aging in yeast (Burtner et al., 2009). Next, we used our competitive-aging approach to measure the CLS of an array of 76 knockout strains aged with or without metformin. In brief, we focused on a limited set of randomly selected genes with at least one human homolog. This collection included 22 mutants in genes with a previously reported CLS phenotype in the Saccharomyces Genome Database (phenotype: chronological lifespan) (Cherry et al., 2012). Functional annotation of these genes points to central eukaryotic processes, such as autophagy (*ATG8*, *HSV2*, *VPS4*), mitochondrial function (*LSC2*, *PET112*, *PIM1*, *HAP3*, *MRPS16*, *MRPL9*), translation (*RPL19B*, *RPL23A*), protein glycosylation (*DIE2*, *ALG3*), meiotic assembly (*KIP1*, *CLB5*), metabolic enzymes (*DUG1*, *PFK1*, *HST2*), and nuclear organization (*CHL1*, *HAT1*, *TEL1*, *NUP170*). The complete list of gene-deletions is provided in Supplementary Data S1.

In both nominal SC and SC with metformin, we observed high quantitative correlation of the phenotypes between replicate plates in the same experiment and between two independent experiments (Supplementary Figure S7). We also confirmed that parameter *G* predicted actual growth rates and resulted in a modest correction of *S*, as expected (Supplementary Figure S8). A direct quantitative comparison of CLS phenotypes under both conditions is shown in Figure 3B (see Supplementary Figure S9 for data rescaling). Most samples were found to fall close to the diagonal; namely the phenotypic effect of the knockout relative to WT was similar under both conditions. The phenotypes of 21 of the 76 knockouts (27%) were significantly different when treated with metformin (Supplementary Figure S10; *t*-test, *p* < 0.05). Mutants with significantly different phenotypes are potential gene-drug interactions, especially those instances with large deviations from the expectation. In many cases, we observed that metformin alleviated or even reverted the short-lifespan effects of the gene deletion, with *pep1*Δ, *die2*Δ, and *alg3*Δ being the most extreme instances. In only one mutant, *lsc2*Δ, the drug significantly aggravated the relative short-lived gene-deletion phenotype. On the opposite scenario, the nominal long-lived phenotype of certain mutants was rendered neutral or closer to neutral with the metformin treatment (e.g., *hsv2*Δ, *ubp13*Δ, and *ubp5*Δ).

We aimed to validate some of the potential gene-drug interactions identified in our screen, focusing on eight mutants with large deviation from the expectation. First, we measured the CLS of wild-type and gene-deletion strains with or without metformin, using the outgrowth OD-kinetics approach (Supplementary Figure S10). A clear effect of metformin treatment on the CLS phenotype was evident for *pep1*Δ, *die2*Δ, and *alg3*Δ; in these cases the short-lifespan phenotype of the mutant was suppressed or alleviated by metformin. We carried out live/dead staining of stationary-phase cells on these three strains, which confirmed gene-drug interactions (Figure 3C). The identified interactions, with specific quantitative information on the magnitude and sign of the effects, indicate a functional association between protein glycosylation and metformin treatment, providing novel information to understand the mechanisms of longevity by metformin in yeast.

Together, results in this section show that competitive-aging yields accurate and replicable large-scale CLS data in a straight-forward manner to shed light on the mechanisms of pharmacological interventions that extend lifespan.

## Discussion

Genetic analysis of the CLS of budding yeast has led to the genome-wide identification of genes involved in aging; recent efforts have sought to describe interactions between genetic and environmental modulators of the phenotype (Garay et al., 2014; Smith et al., 2016; Campos et al., 2018; Jung et al., 2018). A previous report from our group showed advantages of using a competitive-aging approach, in which fluorescently labeled strains in co-culture provide high resolution in parallel setups (Garay et al., 2014). Competitive-aging has also allowed scoring gene-environment interactions at the genomewide level, specifically interactions with dietary restriction (Campos et al., 2018).

Here, we have presented an optimized model to calculate the relative survivorship of deletion strains, taking into account the possible confounding effects coming from growth-rate differences and systematic batch effects. It is certain that major findings in our previous reports would hold given the relatively mild contribution of the *G* and *C* parameters in most samples (the experimental design of such studies precludes fitting data to the model herein presented). Nonetheless, the optimized procedure herein presented is a more exhaustive description of the actual competitive-aging setup, which could improve the scoring CLS phenotypes in specific cases, particularly strains with strong growth defects or experiments with strong batch effects. In addition, we directly compared the performance of a competitive-aging setup to an established approach (Murakami et al., 2008; Jung et al., 2015). Finally, using this enhanced method and data analysis in a proof-of-principle experiment, we were able to unravel significant gene-drug interactions in an array of gene-deletions strains subjected to the lifespan-extending drug metformin.

Early CLS genome-wide screens were based on large pools of gene deletions followed by molecular-barcode hybridization or sequencing (Fabrizio et al., 2010; Matecic et al., 2010; Gresham et al., 2011). These studies provided important insight into which genetic factors mediate stationary phase survival, such as autophagy, vacuolar protein sorting, and regulation of translation. However, the high rates of false positives—specially in the cases of long-lived phenotypes—and low overlap among the sets of genes from different studies (Smith et al., 2016) suggest that systematic errors in barcode detection or major experimental batch effects result in poor experimental replicability. On the other side of the spectrum, an ingenious outgrowth-kinetics approach of yeast monocultures increases the feasibility of percent-survivorship estimates, compared to the conventional colony-forming units method (Murakami et al., 2008); but throughput is still limited with this approach. To overcome this limitation, Jung and co-workers scaled-up this strategy using monocultures in multi-well plates, whereby more strains can be tested in parallel (Jung et al., 2015, 2018). There is still the issue that mild environmental variation can affect separate cultures differently, that could lead to low reproducibility (Burtner et al., 2011); for instance, strains may reach stationary phase at different times after inoculation. In this regards, competitive-aging provides a direct phenotypic comparison and, arguably, more consistent results, given that the mutant population of interest is aged with an internal reference strain under the exact same microenvironment. Importantly, competitive-aging can also be carried out in multi-well plates, enabling high-throughput experimental setups.

One of the inherent drawbacks of the competitive-aging method is that no absolute death-rate information is provided, which can be easily solved through characterization of the wild-type strain in monoculture, as we have shown. Likewise, the dynamic range of this method depends on the actual death rate of the reference strain used. For instance, quantitative phenotypic descriptions are limited if *S* >> 0; in other words only semi-quantitative estimates are possible for extremely long-lived strains (*S*≈*r*_*wt*_). The use of different reference strains, e.g., specific gene deletions with known long-lifespan phenotypes, may help to overcome this limitation. Using different reference strains has allowed to verify that interactions between gene-deletion strains are not very frequent (Campos et al., 2018). Yet, interactions between specific gene-deletion strains and references could take place, for instance when strains release nutrients, toxic or protective compounds to the medium, for which competing strains could differentially affected in terms of survivorship (Perrone et al., 2005; Santos et al., 2012, 2015). We also note that differences in the signal dynamics of the fluorophores used may result in systematically biased quantifications, which could be overcome by including mutants labeled with different fluorescent proteins or dye-swap experiments. Here, we used a simple exponential function to model cell-viability decay in the population; another potential improvement would be to test functions with additional parameters that could better model survivorship in stationary phase, such as the Gompertz function (Qin and Lu, 2006). Finally, just as in most population-based CLS methods, competitive aging depends on cells being able to re-enter the cell cycle, which can only be distinguished from actual death using outgrowth-independent methods, such as live/dead staining.

We have illustrated the potential of competitive-aging screening by characterizing an array of yeast deletion strains exposed to metformin. A number of lifespan-extending pharmacological interventions are already being tested for age-related diseases in humans, even when the mechanisms underlying their beneficial effects are frequently not fully understood (Mullard, 2018; Zimmermann et al., 2018). Given that lifespan is a complex phenotype, identifying conserved gene-drug interactions could shed light on the modes of action and to pinpoint genetic modifiers of the drug’s effects (Zimmermann et al., 2018). By screening an array of 76 gene deletions aged with metformin, we found a number of cases in which metformin buffers both short- or long-lifespan mutant phenotypes. The interacting genes suggest a role of protein glycosylation and protein homeostasis, which is in line with previous evidence showing that metformin alters glycation and protein transport in yeast (Kazi et al., 2017; Stynen et al., 2018). Our results also uncovered interactions between metformin and genes involved in mitochondrial function (*erp6*Δ, *lsc2*Δ, *ema35*Δ, and *ylh47*Δ), which is a known player in the cellular response to metformin (Borklu-Yucel et al., 2015; Stynen et al., 2018). It must be noted that reduced acidification of aging medium by metformin—either by reduced secretion of acetic acid or direct media buffering—could explain part of the increased survivorship of the wild-type strain, given that acetic-acid toxicity is a mechanisms of chronological aging in yeast (Burtner et al., 2009). This should also be taken into account while interpreting specific gene-drug interaction of genes involved in protein glycosylation, or other hits involving mutants that are sensitive to acid, such as *pep1*Δ (Hoepfner et al., 2014). Specific mechanisms involving increased resistance to acidic conditions remain to be addressed, along with the possible interaction of protein glycosylation with the mitochondrial electron transport chain and homeostasis of copper and iron (Logie et al., 2012; Stynen et al., 2018), which are already known to participate in the response to metformin.

Competitive-aging can readily be adapted to screen double mutants at large scale and to score genetic interactions underlying CLS phenotypes. Genetic interactions (epistasis) are a powerful tool to describe the architecture of phenotypes and the functional relationships of different genetic pathways (Segrè et al., 2005; Collins et al., 2007; Onge et al., 2007; Phillips, 2008; Kuzmin et al., 2018). While epistasis-network analyses in yeast have granted deep knowledge of the genetic landscape of mitotically active, proliferating cells, less is known about how genetic interactions shape the genetic architecture of post-mitotic survivorship. Large-scale genetic analysis of double-mutants aged in competition with their single-mutant references is an attractive experimental setup to identify interactions among different genes and pathways underlying CLS in yeast. Our competitive-aging screening method and quantitative analysis provide a powerful systematic tool to shed light on the complex genetic, environmental, and pharmacological wiring of aging cells.

## Data Availability Statement

All datasets generated for this study are included in the article/Supplementary Material.

## Author Contributions

JA-R and AD conceived and designed the study and wrote the manuscript. JA-R, MM-F, AJ-R, and SC performed the experiments. JA-R, EG, and NS developed and implemented the model. JA-R, SC, and AD analyzed the data. AD acquired funding. All authors read and approved the final version of the submitted manuscript.

## Conflict of Interest

The authors declare that the research was conducted in the absence of any commercial or financial relationships that could be construed as a potential conflict of interest.

## Supplementary Material

FIGURE S1 Examples of raw data for *OD*_600_, and *RFPraw* and *CFPraw* signal from outgrowth-culture kinetics monitored throughout the experiment.

FIGURE S2 Multiple linear modeling fits relative survivorship and relative growth rate from competitive-aging cultures.

FIGURE S3 Comparing replicability of CLS measurements from two methods.

FIGURE S4 Variation in *S* decreases with the number of reference samples used.

FIGURE S5 The parameter *G* corrects effects of differential growth rates that otherwise affect relative survivorship, *S*.

FIGURE S6 Metformin limits media acidification in stationary phase.

FIGURE S7 Relative survivorship (S) is well replicated between plates within the same experimental batch, as well as between different batches.

FIGURE S8 The parameter *G* corrects effects of differential growth rates that otherwise affect relative survivorship, *S*.

FIGURE S9 Relative survivorship (*S*) data rescaling.

FIGURE S10 Candidate gene-drug interactions and validation.

TABLE S1 Recipes for media used in this study.

NOTE S1 Development and implementation of a linear model to calculate relative survivorship of mutant strains in competitive-aging cultures.

DATA S1 Array of mutants used for gene-drug interactions, with their relative survivorship, rescaled survivorship, and relative growth rates.

## References

[B1] Alvers A. L. Fishwick L. K. Wood M. S. Hu D. Chung H. S. Dunn W. A. Jr. (2009). Autophagy and amino acid homeostasis are required for chronological longevity in *Saccharomyces cerevisiae*. *Aging Cell* 8 353–369. 10.1111/j.1474-9726.2009.00469.x 19302372 PMC2802268

[B2] Amberg D. C. (2005). *Methods in Yeast Genetics: a Cold Spring Harbor Laboratory Course Manual.* New York, NY: Cold Spring Harbor Laboratory Press.

[B3] Avelar-Rivas J. A. Munguía-Figueroa M. Juárez-Reyes A. Garay E. Campos S. E. Shoresh N. (2020). A competitive-aging method for quantitative genetic analysis of the chronological lifespan of Saccharomyces cerevisiae. *bioRxiv* [Preprint]. 10.1101/696682 PMC7240105 32477409

[B4] Barre B. Hallin J. Yue J.-X. Persson K. Mikhalev E. Irizar A. (2020). Intragenic repeat expansion in the cell wall protein gene *HPF1* controls yeast chronological aging. *Genome Res.* 10:gr.253351.119. 10.1101/gr.253351.119 32277013 PMC7263189

[B5] Borklu-Yucel E. Eraslan S. Ulgen K. O. (2015). Transcriptional remodeling in response to transfer upon carbon-limited or metformin-supplemented media in *S. cerevisiae* and its effect on chronological life span. *Appl. Microbiol. Biotechnol.* 99 6775–6789. 10.1007/s00253-015-6728-5 26099330

[B6] Burtner C. R. Murakami C. J. Kennedy B. K. Kaeberlein M. (2009). A molecular mechanism of chronological aging in yeast. *Cell Cycle* 8 1256–1270. 10.4161/cc.8.8.8287 19305133 PMC2746416

[B7] Burtner C. R. Murakami C. J. Olsen B. Kennedy B. K. Kaeberlein M. (2011). A genomic analysis of chronological longevity factors in budding yeast. *Cell Cycle* 10 1385–1396. 10.4161/cc.10.9.15464 21447998 PMC3356828

[B8] Campisi J. Kapahi P. Lithgow G. J. Melov S. Newman J. C. Verdin E. (2019). From discoveries in ageing research to therapeutics for healthy ageing. *Nature* 571, 183–192. 10.1038/s41586-019-1365-2 31292558 PMC7205183

[B9] Campos S. E. Avelar-Rivas J. A. Garay E. Juárez-Reyes A. DeLuna A. (2018). Genomewide mechanisms of chronological longevity by dietary restriction in budding yeast. *Aging Cell* 17:e12749. 10.1111/acel.12749 29575540 PMC5946063

[B10] Campos S. E. DeLuna A. (2019). Functional genomics of dietary restriction and longevity in yeast. *Mech. Ageing Dev.* 179 36–43. 10.1016/J.MAD.2019.02.003 30790575

[B11] Cherry J. M. Hong E. L. Amundsen C. Balakrishnan R. Binkley G. Chan E. T. (2012). Saccharomyces genome database: the genomics resource of budding yeast. *Nucleic Acids Res.* 40 D700–D705. 10.1093/nar/gkr1029 22110037 PMC3245034

[B12] Collins S. R. Miller K. M. Maas N. L. Roguev A. Fillingham J. Chu C. S. (2007). Functional dissection of protein complexes involved in yeast chromosome biology using a genetic interaction map. *Nature* 446 806–810. 10.1038/nature05649 17314980

[B13] Eisenberg T. Knauer H. Schauer A. Büttner S. Ruckenstuhl C. Carmona-Gutierrez D. (2009). Induction of autophagy by spermidine promotes longevity. *Nat. Cell Biol.* 11 1305–1314. 10.1038/ncb1975 19801973

[B14] Fabrizio P. Hoon S. Shamalnasab M. Galbani A. Wei M. Giaever G. (2010). Genome-wide screen in *Saccharomyces cerevisiae* identifies vacuolar protein sorting, autophagy, biosynthetic, and tRNA methylation genes involved in life span regulation. *PLoS Genet.* 6:e1001024. 10.1371/journal.pgen.1001024 20657825 PMC2904796

[B15] Fabrizio P. Longo V. D. (2003). The chronological life span of *Saccharomyces cerevisiae*. *Aging Cell* 2 73–81. 10.1046/j.1474-9728.2003.00033.x 12882320

[B16] Fabrizio P. Pozza F. Pletcher S. D. Gendron C. M. Longo V. D. (2001). Regulation of longevity and stress resistance by Sch9 in yeast. *Science* 292 288–290. 10.1126/science.1059497 11292860

[B17] Garay E. Campos S. E. González de la Cruz J. Gaspar A. P. Jinich A. DeLuna A. (2014). High-resolution profiling of stationary-phase survival reveals yeast longevity factors and their genetic interactions. *PLoS Genet.* 10:e1004168. 10.1371/journal.pgen.1004168 24586198 PMC3937222

[B18] Gems D. Partridge L. (2013). Genetics of longevity in model organisms: debates and paradigm shifts. *Annu. Rev. Physiol.* 75 621–644. 10.1146/annurev-physiol-030212-183712 23190075

[B19] Gresham D. Boer V. M. Caudy A. Ziv N. Brandt N. J. Storey J. D. (2011). System-level analysis of genes and functions affecting survival during nutrient starvation in *Saccharomyces cerevisiae*. *Genetics* 187 299–317. 10.1534/genetics.110.120766 20944018 PMC3018308

[B20] Hoepfner D. Helliwell S. B. Sadlish H. Schuierer S. Filipuzzi I. Brachat S. (2014). High-resolution chemical dissection of a model eukaryote reveals targets, pathways and gene functions. *Microbiol. Res.* 169 107–120. 10.1016/j.micres.2013.11.004 24360837

[B21] Hu J. Wei M. Mirisola M. G. Longo V. D. (2013). *Assessing Chronological Aging in Saccharomyces Cerevisiae.* Totowa, NJ: Humana Press, 463–472. 10.1007/978-1-62703-239-1_30 PMC4041521 23296677

[B22] Jung P. P. Christian N. Kay D. P. Skupin A. Linster C. L. (2015). Protocols and programs for high-throughput growth and aging phenotyping in yeast. *PLoS One* 10:e0119807. 10.1371/journal.pone.0119807 25822370 PMC4379057

[B23] Jung P. P. Zhang Z. Paczia N. Jaeger C. Ignac T. May P. (2018). Natural variation of chronological aging in the *Saccharomyces cerevisiae* species reveals diet-dependent mechanisms of life span control. *Aging Mech. Dis.* 4:3. 10.1038/s41514-018-0022-6 29560271 PMC5845861

[B24] Kazi R. S. Banarjee R. M. Deshmukh A. B. Patil G. V. Jagadeeshaprasad M. G. Kulkarni M. J. (2017). Glycation inhibitors extend yeast chronological lifespan by reducing advanced glycation end products and by back regulation of proteins involved in mitochondrial respiration. *J. Proteomics* 156 104–112. 10.1016/J.JPROT.2017.01.015 28132874

[B25] Kirkwood T. B. L. (2008). A systematic look at an old problem. *Nature* 451 644–647. 10.1038/451644a 18256658

[B26] Kuzmin E. VanderSluis B. Wang W. Tan G. Deshpande R. Chen Y. (2018). Systematic analysis of complex genetic interactions. *Science* 360:eaao1729. 10.1126/science.aao1729 29674565 PMC6215713

[B27] Laschober G. T. Ruli D. Hofer E. Muck C. Carmona-Gutierrez D. Ring J. (2010). Identification of evolutionarily conserved genetic regulators of cellular aging. *Aging Cell* 9 1084–1097. 10.1111/j.1474-9726.2010.00637.x 20883526 PMC2997327

[B28] Logie L. Harthill J. Patel K. Bacon S. Hamilton D. L. Macrae K. (2012). Cellular responses to the metal-binding properties of metformin. *Diabetes* 61 1423–1433. 10.2337/db11-0961 22492524 PMC3357267

[B29] Longo V. D. Fabrizio P. (2012). Chronological aging in *Saccharomyces cerevisiae*. *Subcell. Biochem.* 57 101–121. 10.1007/978-94-007-2561-4_5 22094419 PMC4048025

[B30] Longo V. D. Shadel G. S. Kaeberlein M. Kennedy B. (2012). Replicative and chronological aging *in Saccharomyces cerevisiae*. *Cell Metab.* 16 18–31. 10.1016/J.CMET.2012.06.002 22768836 PMC3392685

[B31] López-Otín C. Blasco M. A. Partridge L. Serrano M. Kroemer G. (2013). The hallmarks of aging. *Cell* 153 1194–1217. 10.1016/J.CELL.2013.05.039 23746838 PMC3836174

[B32] Matecic M. Smith D. L. Pan X. Maqani N. Bekiranov S. Boeke J. D. (2010). A microarray-based genetic screen for yeast chronological aging factors. *PLoS Genet.* 6:e1000921. 10.1371/journal.pgen.1000921 20421943 PMC2858703

[B33] Mullard A. (2018). Anti-ageing pipeline starts to mature. *Nat. Rev. Drug Discov.* 17 609–612. 10.1038/nrd.2018.134 30072728

[B34] Murakami C. J. Burtner C. R. Kennedy B. K. Kaeberlein M. (2008). A method for high-throughput quantitative analysis of yeast chronological life span. *J. Gerontol. Ser. A Biol. Sci. Med. Sci.* 63 113–121. 10.1093/gerona/63.2.113 18314444

[B35] Onge R. P. S. Mani R. Oh J. Proctor M. Fung E. Davis R. W. (2007). Systematic pathway analysis using high-resolution fitness profiling of combinatorial gene deletions. *Nat. Genet.* 39 199–206. 10.1038/ng1948 17206143 PMC2716756

[B36] Perrone G. G. Grant C. M. Dawes I. W. (2005). Genetic and environmental factors influencing glutathione homeostasis in *Saccharomyces cerevisiae*. *Mol. Biol. Cell* 16 218–230. 10.1091/mbc.E04-07-0560 15509654 PMC539166

[B37] Phillips P. C. (2008). Epistasis — the essential role of gene interactions in the structure and evolution of genetic systems. *Nat. Rev. Genet.* 9 855–867. 10.1038/nrg2452 18852697 PMC2689140

[B38] Powers R. W. Kaeberlein M. Caldwell S. D. Kennedy B. K. Fields S. Fields S. (2006). Extension of chronological life span in yeast by decreased TOR pathway signaling. *Genes Dev.* 20 174–184. 10.1101/gad.1381406 16418483 PMC1356109

[B39] Qin H. Lu M. (2006). Natural variation in replicative and chronological life spans of *Saccharomyces cerevisiae*. *Exp. Gerontol.* 41 448–456. 10.1016/J.EXGER.2006.01.007 16516427

[B40] Santos J. Leão C. Sousa M. J. (2012). Growth culture conditions and nutrient signaling modulating yeast chronological longevity. *Oxid. Med. Cell. Longev.* 2012 1–10. 10.1155/2012/680304 22928083 PMC3425870

[B41] Santos J. Leitão-Correia F. Sousa M. J. Leão C. (2015). Ammonium is a key determinant on the dietary restriction of yeast chronological aging in culture medium. *Oncotarget* 6 6511–6523. 10.18632/oncotarget.2989 25576917 PMC4466630

[B42] Schroeder E. A. Raimundo N. Shadel G. S. (2013). Epigenetic silencing mediates mitochondria stress-induced longevity. *Cell Metab.* 17 954–964. 10.1016/j.cmet.2013.04.003 23747251 PMC3694503

[B43] Segrè D. DeLuna A. Church G. M. Kishony R. (2005). Modular epistasis in yeast metabolism. *Nat. Genet.* 37 77–83. 10.1038/ng1489 15592468

[B44] Sheff M. A. Thorn K. S. (2004). Optimized cassettes for fluorescent protein tagging in *Saccharomyces cerevisiae*. *Yeast* 21 661–670. 10.1002/yea.1130 15197731

[B45] Smith D. L. Maharrey C. H. Carey C. R. White R. A. Hartman J. L. Hartman J. L. (2016). Gene-nutrient interaction markedly influences yeast chronological lifespan. *Exp. Gerontol.* 86 113–123. 10.1016/j.exger.2016.04.012 27125759 PMC5079838

[B46] Stynen B. Abd-Rabbo D. Kowarzyk J. Miller-Fleming L. Aulakh S. K. Garneau P. (2018). Changes of cell biochemical states are revealed in protein homomeric complex dynamics. *Cell* 175 1418.e9–1429.e9. 10.1016/J.CELL.2018.09.050 30454649 PMC6242466

[B47] Wei M. Fabrizio P. Hu J. Ge H. Cheng C. Li L. (2008). Life span extension by calorie restriction depends on Rim15 and transcription factors downstream of Ras/PKA, Tor, and Sch9. *PLoS Genet.* 4:e13. 10.1371/journal.pgen.0040013 18225956 PMC2213705

[B48] Zimmermann A. Hofer S. Pendl T. Kainz K. Madeo F. Carmona-Gutierrez D. (2018). Yeast as a tool to identify anti-aging compounds. *FEMS Yeast Res.* 18:foy020. 10.1093/femsyr/foy020 29905792 PMC6001894

