# Supplementary material for: An Optimized Competitive-Aging Method Reveals Gene-Drug Interactions Underlying the Chronological Lifespan of Saccharomyces cerevisiae
Source: Front Genet. 2020 May 14;11:468. doi: 10.3389/fgene.2020.00468 (PMC7240105; doi:10.3389/fgene.2020.00468)
Supplement: FIGURE S1 — Examples of raw data for OD600, and RFPraw and CFPraw signal from outgrowth-culture kinetics monitored throughout the experiment. [file Data_Sheet_1.zip › 01-AVELAR_NoteS1.pdf]

Supplementary Note 1. Development and implementation of a linear model to calculate relative survival of mutant strains in competitive-aging cultures

To obtain relative survivorship of competitive-aging cultures we modeled WT and mutant populations with initial sizes $N_{wt_{T0}}$ and $N_{x_{T0}}$ and declining trough time in stationary phase at constant exponential rates r_{wt} and r_x. After T_i days, the sizes of the remaining viable populations are $N_{wt_{T_i}} = N_{wtT_0} \cdot e^{-r_{wt} \cdot T_i}$ and $N_{x_{T_i}} = N_{xT_0} \cdot e^{-r_x T_i}$. In every outgrowth sampling, we inoculated small volumes of this aged co-culture onto fresh medium, in which both populations resume cell division and grow exponentially at rates g_{wt} and g_x. At any outgrowth, the population sizes after t_j hours are $N_{wt_{Tt_j}} = N_{wt_T} \cdot e^{g_{wt} \cdot t_j}$ and $N_{x_{Tt_j}} = N_{x_T} \cdot e^{g_x \cdot t_j}$. Given that both strains are aged and grown in co-culture, the relationship between the sizes of their populations at any time, t_j, during the outgrowth T_i is:

$$ln\ (\frac{N_x}{N_{wt}})\ _{T_i,\, t_j} = ln\ (\frac{N_x}{N_{wt}})\ _{T_0} + (r_{wt} - r_x)T_i + (g_x - g_{wt})(t_j)$$

As shown in Figure S1, the measurements of *CFP* and *RFP* signals are proportional to $N_{wt_{Tt}}$ and $N_{x_{Tt}}$, therefore, the experimental measurements of each well, $w,$ are equal to the linear equation:

$$ln\ (\frac{RFP}{CFP})\ _{T_i,\, t_j} = A_w + S_w \cdot T_i + G_w \cdot t_j + C_{T_i,\, t_j}$$

We run a multiple linear regression where we fit the data obtained from measuring bulk RFP signal (*RFP*), and bulk CFP signal (*CFP*) in the outgrowth cultures in fresh medium sampled by inoculating from aging stationary phase co-cultures. The first step is to subtract auto-fluorescence RFP_{raw} from WT_{CFP} as a function of the OD_{600} from all RFP_{raw} measurements; the same is done for CFP_{raw} from WT_{RFP}. We keep outgrowth measurements for days in which we observe an increase in both RFP_{raw} and CFP_{raw}. This leaves us with the *RFP* and *CFP* quantities that should be a function of the death and growth rates (r and g). For each measured well, w, we

expressed such relationship using the model $ln\left(\frac{RFP}{CFP}\right)_{T_i,t_j} = A_w + S_w \cdot T_i + G_w \cdot t_j + C_{T_i,t_j}$ in which A_w reflects the proportion $ln\left(\frac{RFP}{CFP}\right)_{T_0,t_0}$ at the beginning of the experiment, this number will depend on the amount of Δx_{RFP} and WT_{CFP} strains that are mixed together when the aging co-cultures are set up. The relative survivorship is modeled in $S_w = (r_{wt} - r_x)$, which is expressed in terms of the number of the time T (in days) that the co-culture has been in stationary phase. Meanwhile the difference in growth rates of both strains is modeled in $G_w = (g_x - g_{wt})$, which is expressed in terms of the time t (in hours) within every outgrowth. Finally, the model takes into account the batch error of each plate read, as C_{T_i,t_j}.

To fit the data, we generate a system of linear equations. For well w_1, there are as many equations as the total number of measurements of that well across the entire experiment. For instance, day number one, T_1, has the following equations:

*Eq*1.1.1 $$ln\left(\frac{RFP_{w_1}}{CFP_{w_1}}\right)_{T_1,t_1} = A_{w_1} + S_{w_1} \cdot T_1 + G_{w_1} \cdot t_{1.1} + C_{T_1,t_1}$$

*Eq*1.2.1 $$ln\left(\frac{RFP_{w_1}}{CFP_{w_1}}\right)_{T_1,t_2} = A_{w_1} + S_{w_1} \cdot T_1 + G_{w_1} \cdot t_{1.2} + C_{T_1,t_2}$$

*Eq*1.3.1 $$ln\left(\frac{RFP_{w_1}}{CFP_{w_1}}\right)_{T_1,t_3} = A_{w_1} + S_{w_1} \cdot T_1 + G_{w_1} \cdot t_{1.3} + C_{T_1,t_3}$$

*Eq*1.*m*.1 $$ln\left(\frac{RFP_{w_1}}{CFP_{w_1}}\right)_{T_1,t_m} = A_{w_1} + S_{w_1} \cdot T_1 + G_{w_1} \cdot t_{1.m} + C_{T_1,t_m}$$

In these equations, the term $ln\left(\frac{RFP_{w_1}}{CFP_{w_1}}\right)_{T_1,t_m}$ is the empirical determination of the logarithmic quotient of both fluorescent signals at each measurement of the outgrowth in exponential phase. A_{w_1} is the same variable for all equations of w_1, likewise for $S_{w_1} \cdot T_1$ because the relative death rate of the well (S_{w_1}) is the same and is multiplied by the same number of days of the experiment, T_1. By definition, the first day of measurement is considered time $T_1 = 0$ for all plates and wells. One of the two terms that change between these equations is, $G_{w_1} \cdot t_{1.m}$. Although the

relative growth rate is the same for each well throughout the entire experiment, the time at which the measurement is done changes and that is reflected in the different coefficients that these equations will get for those terms. For example, if measurements are done every 1.5 hours and the exponential phase starts at the third measurement, coefficients would be $t_{1.1} = 3$; $t_{1.2} = 4.5$; $t_{1.3} = 6$. Finally, the other term that changes in these equations is C_{T_1,t_m} which reflects that there are batch effects influencing measurements; such effects appear any time a plate is read, and so it has similar effects in all of the wells within the same plate. For that reason C_{T_1,t_m} is different at each measurement of a well, but is shared in all wells from the same plate read. Therefore, the measurements of different wells in one plate with k wells lead to equations:

*Eq*1.1.1 $$ln\left(\frac{RFP_{w_1}}{CFP_{w_1}}\right)_{T_1,t_1} = A_{w_1} + S_{w_1} \cdot T_1 + G_{w_1} \cdot t_{1.1} + C_{T_1,t_1}$$

*Eq*1.1.2 $$ln\left(\frac{RFP_{w_2}}{CFP_{w_2}}\right)_{T_1,t_1} = A_{w_2} + S_{w_2} \cdot T_1 + G_{w_2} \cdot t_{1.1} + C_{T_1,t_1}$$

*Eq*1.1.3 $$ln\left(\frac{RFP_{w_3}}{CFP_{w_3}}\right)_{T_1,t_1} = A_{w_3} + S_{w_3} \cdot T_1 + G_{w_3} \cdot t_{1.1} + C_{T_1,t_1}$$

*Eq*1.1.*k* $$ln\left(\frac{RFP_{w_k}}{CFP_{w_k}}\right)_{T_1,t_1} = A_{w_k} + S_{w_k} \cdot T_1 + G_{w_k} \cdot t_{1.1} + C_{T_1,t_1}$$

The same wells measured the second day will have the equations:

*Eq*2.1.1 $$ln\left(\frac{RFP_{w_1}}{CFP_{w_1}}\right)_{T_2,t_1} = A_{w_1} + S_{w_1} \cdot T_2 + G_{w_1} \cdot t_{2.1} + C_{T_2,t_1}$$

*Eq*2.1.2 $$ln\left(\frac{RFP_{w_2}}{CFP_{w_2}}\right)_{T_2,t_1} = A_{w_2} + S_{w_2} \cdot T_2 + G_{w_2} \cdot t_{2.1} + C_{T_2,t_1}$$

*Eq*2.1.1 $$ln\left(\frac{RFP_{w_3}}{CFP_{w_3}}\right)_{T_2,t_1} = A_{w_3} + S_{w_3} \cdot T_2 + G_{w_3} \cdot t_{2.1} + C_{T_2,t_1}$$

*Eq*2.1.*k* $$ln\left(\frac{RFP_{w_k}}{CFP_{w_k}}\right)_{T_2,t_1} = A_{w_k} + S_{w_k} \cdot T_2 + G_{w_k} \cdot t_{2.1} + C_{T_2,t_1}$$

If the second outgrowth occurs after one day then $T_2 = 1$. It is worth pointing out that this $t_{2.1}$ is different from $t_{1.1}$ because the outgrowths of each day are not measured at identical points in time. For example, it could be that $t_{2.1} = 3.6$ and $t_{2.2} = 4.1$.

We verified the quality of the data and set some restrictions. For instance, in every valid measurement both fluorescent signals must be above the maximum auto-fluorescence detection, as indicated by wells with monocultures of WT_{CFP} or WT_{RFP} strain with auto-fluorescence of the opposite signal. This step removes all monoculture wells from the system of equations.

Our model fitting has the assumption that WT_{CFP}+WT_{RFP} competitions have $S_x = 0$ and $G_x = 0$, so deviations from such values in wells ref, containing such mixture of strains depend on the well specific variable $A_{w_{ref.i}}$or to the plate variables C_{T_n,t_m}. To accomplish that, we eliminate S and G from the reference-well equations, so every measurement from those wells will feed the system of equations as follows:

*Eq*1.*r*.1 $$ln\left(\frac{RFP_{w_{ref.i}}}{CFP_{ref.i}}\right)_{T_1,t_1} = A_{w_{ref.i}} + C_{T_1,t_1}$$

*Eq*1.*r*.2 $$ln\left(\frac{RFP_{ref.i}}{CFP_{w_{ref.i}}}\right)_{T_1,t_2} = A_{w_{ref.i}} + C_{T_1,t_2}$$

*Eq*2.*r*.1 $$ln\left(\frac{RFP_{w_{ref.i}}}{CFP_{w_{ref.i}}}\right)_{T_2,t_1} = A_{w_{ref.i}} + C_{T_2,t_1}$$

To find an approximation to the solution of the entire set of equations, we built a predictor matrix that contains all the valid measurements of the plate and the logarithmic ratio of the actual experimental measurements as the responses vector. In this example, we show the matrix for wells w_1,w_2, w_{ref} and w_k outgrown at days T_1, T_2 and T_n with 3 measurements per outgrowth $t_{n.1}$, $t_{n.2}$ and $t_{n.m}$:

Aw_1	Sw_1	Gw_1	Aw_2	Sw_2	Gw_2	Aw_{ref}	Aw_k	Sw_k	Gw_k	CT_1t_1	CT_1t_2	CT_1t_m	CT_2t_1	CT_2t_2	CT_2t_m	CT_nt_1	CT_nt_2	CT_nt_m
1	T_1	$t_{1.1}$	0	0	0	0	0	0	0	1	0	0	0	0	0	0	0	0
1	T_1	$t_{1.2}$	0	0	0	0	0	0	0	0	1	0	0	0	0	0	0	0
1	T_1	$t_{1.m}$	0	0	0	0	0	0	0	0	0	1	0	0	0	0	0	0
1	T_2	$t_{2.1}$	0	0	0	0	0	0	0	0	0	0	1	0	0	0	0	0
1	T_2	$t_{2.2}$	0	0	0	0	0	0	0	0	0	0	0	1	0	0	0	0
1	T_2	$t_{2.m}$	0	0	0	0	0	0	0	0	0	0	0	0	1	0	0	0
1	T_n	$t_{n.1}$	0	0	0	0	0	0	0	0	0	0	0	0	0	1	0	0
1	T_n	$t_{n.2}$	0	0	0	0	0	0	0	0	0	0	0	0	0	0	1	0
1	T_n	$t_{n.m}$	0	0	0	0	0	0	0	0	0	0	0	0	0	0	0	1
0	0	0	1	T_1	$t_{1.1}$	0	0	0	0	1	0	0	0	0	0	0	0	0
0	0	0	1	T_1	$t_{1.2}$	0	0	0	0	0	1	0	0	0	0	0	0	0
0	0	0	1	T_1	$t_{1.m}$	0	0	0	0	0	0	1	0	0	0	0	0	0
0	0	0	1	T_2	$t_{2.1}$	0	0	0	0	0	0	0	1	0	0	0	0	0
0	0	0	1	T_2	$t_{2.2}$	0	0	0	0	0	0	0	0	1	0	0	0	0
0	0	0	1	T_2	$t_{2.m}$	0	0	0	0	0	0	0	0	0	1	0	0	0
0	0	0	1	T_n	$t_{n.1}$	0	0	0	0	0	0	0	0	0	0	1	0	0
0	0	0	1	T_n	$t_{n.2}$	0	0	0	0	0	0	0	0	0	0	0	1	0
0	0	0	1	T_n	$t_{n.m}$	0	0	0	0	0	0	0	0	0	0	0	0	1
0	0	0	0	0	0	1	0	0	0	1	0	0	0	0	0	0	0	0
0	0	0	0	0	0	1	0	0	0	0	1	0	0	0	0	0	0	0
0	0	0	0	0	0	1	0	0	0	0	0	1	0	0	0	0	0	0
0	0	0	0	0	0	1	0	0	0	0	0	0	1	0	0	0	0	0
0	0	0	0	0	0	1	0	0	0	0	0	0	0	1	0	0	0	0
0	0	0	0	0	0	1	0	0	0	0	0	0	0	0	1	0	0	0
0	0	0	0	0	0	1	0	0	0	0	0	0	0	0	0	1	0	0
0	0	0	0	0	0	1	0	0	0	0	0	0	0	0	0	0	1	0
0	0	0	0	0	0	1	0	0	0	0	0	0	0	0	0	0	0	1
0	0	0	0	0	0	0	1	T_1	$t_{1.1}$	1	0	0	0	0	0	0	0	0
0	0	0	0	0	0	0	1	T_1	$t_{1.2}$	0	1	0	0	0	0	0	0	0
0	0	0	0	0	0	0	1	T_1	$t_{1.m}$	0	0	1	0	0	0	0	0	0
0	0	0	0	0	0	0	1	T_2	$t_{2.1}$	0	0	0	1	0	0	0	0	0
0	0	0	0	0	0	0	1	T_2	$t_{2.2}$	0	0	0	0	1	0	0	0	0
0	0	0	0	0	0	0	1	T_2	$t_{2.m}$	0	0	0	0	0	1	0	0	0
0	0	0	0	0	0	0	1	T_n	$t_{n.1}$	0	0	0	0	0	0	1	0	0
0	0	0	0	0	0	0	1	T_n	$t_{n.2}$	0	0	0	0	0	0	0	1	0
0	0	0	0	0	0	0	1	T_n	$t_{n.m}$	0	0	0	0	0	0	0	0	1

If there are k wells in a plate, the plate was measured $m*n$ times then there are $k*m*n$ rows in the matrix and there are $3k+m*n-2Nw_{ref}$ variables (or columns)

to fit (Nw_{ref} is the number of wells that contain reference co-cultures); hence, for the previous matrix there are 36 rows and 19 predictor variables.

We applied a multiple linear regression to approximate the solution. The Matlab function *regress* was used, as it provides useful statistics about the fit such as the 95% confidence interval for the coefficient estimates.

Scripts and documentation to run this model are available in GitHub: https://abrahamavelar.github.io/LinearModelCLS/
