# Supplementary material for: An Optimized Competitive-Aging Method Reveals Gene-Drug Interactions Underlying the Chronological Lifespan of Saccharomyces cerevisiae
Source: Front Genet. 2020 May 14;11:468. doi: 10.3389/fgene.2020.00468 (PMC7240105; doi:10.3389/fgene.2020.00468)
Supplement: FIGURE S1 — Examples of raw data for OD600, and RFPraw and CFPraw signal from outgrowth-culture kinetics monitored throughout the experiment. [file Data_Sheet_1.zip › 02-AVELAR_TableS1.pdf]

Table S1. Media recipes

Aging medium (SC)

Reagents
Difco™ Yeast Nitrogen Base w/o Amino Acids (ref 291940)
D-(+)- Glucose (Sigma G2870)
Yeast Synthetic Drop-out Medium Supplements **without uracil** (Sigma Y1501)
Uracil (Sigma, U0750)

Recipe
1. Before mixing any of the components 1.79 g of Uracil were added to the commercial Synthetic Drop-out mix (20 g) to obtain a complete amino acid mix
2. For 1 L of SC medium mix together:
3. Add the components to 500 mL of deionized water and dissolve
4. Add deionized water to make 1 L of medium.
5. Filter sterilize

Note: do not buffer pH

YNB low fluorescence medium (YNB-lf)

Recipe
1. For 1 L of YNB-lf medium, mix together:

-5 g	$(NH_4)_2SO_4$
-1 g	KH_2PO_4
- 0.5 g	$MgSO_4$
- 0.1 g	NaCl
- 0.1 g	$CaCl_2$
- 20 g	D-(+)- Glucose (Sigma G2870)
- 0.79 g	CSM (complete supplement mixture, MP)

2. Add the components to 500 mL of deionized water and dissolve thoroughly
3. Add 1 mL of trace element stock*
4. Add 1 mL of vitamin stock**
5. Add deionized water to make 1 L of medium
6. Filter sterilize

***Trace-element stock**
- Mix the indicated amount of components together:

50mg	H_3BO_3
4mg	$CuSO_4$
10mg	KI
20mg	$FeCl_3$
40mg	$MnSO_4$
20mg	Na_2MoO_4
40mg	$ZnSO_4$

- Bring to 100 mL with deionized water

(...)

**** Vitamin stock**

- Mix the indicated amount of components together:

0.2mg	Biotin
40mg	Calcium pantothenate
200mg	Inositol
40mg	Niacin
20mg	Para-amino benzoic acid
40mg	Pyroxidine HCl
40mg	Thiamine HCl

- Bring to 100 mL with deionized water
