# Supplementary material for: An Optimized Competitive-Aging Method Reveals Gene-Drug Interactions Underlying the Chronological Lifespan of Saccharomyces cerevisiae
Source: Front Genet. 2020 May 14;11:468. doi: 10.3389/fgene.2020.00468 (PMC7240105; doi:10.3389/fgene.2020.00468)
Supplement: FIGURE S1 — Examples of raw data for OD600, and RFPraw and CFPraw signal from outgrowth-culture kinetics monitored throughout the experiment. [file Data_Sheet_1.zip › 03-AVELAR_FigS1.pdf]

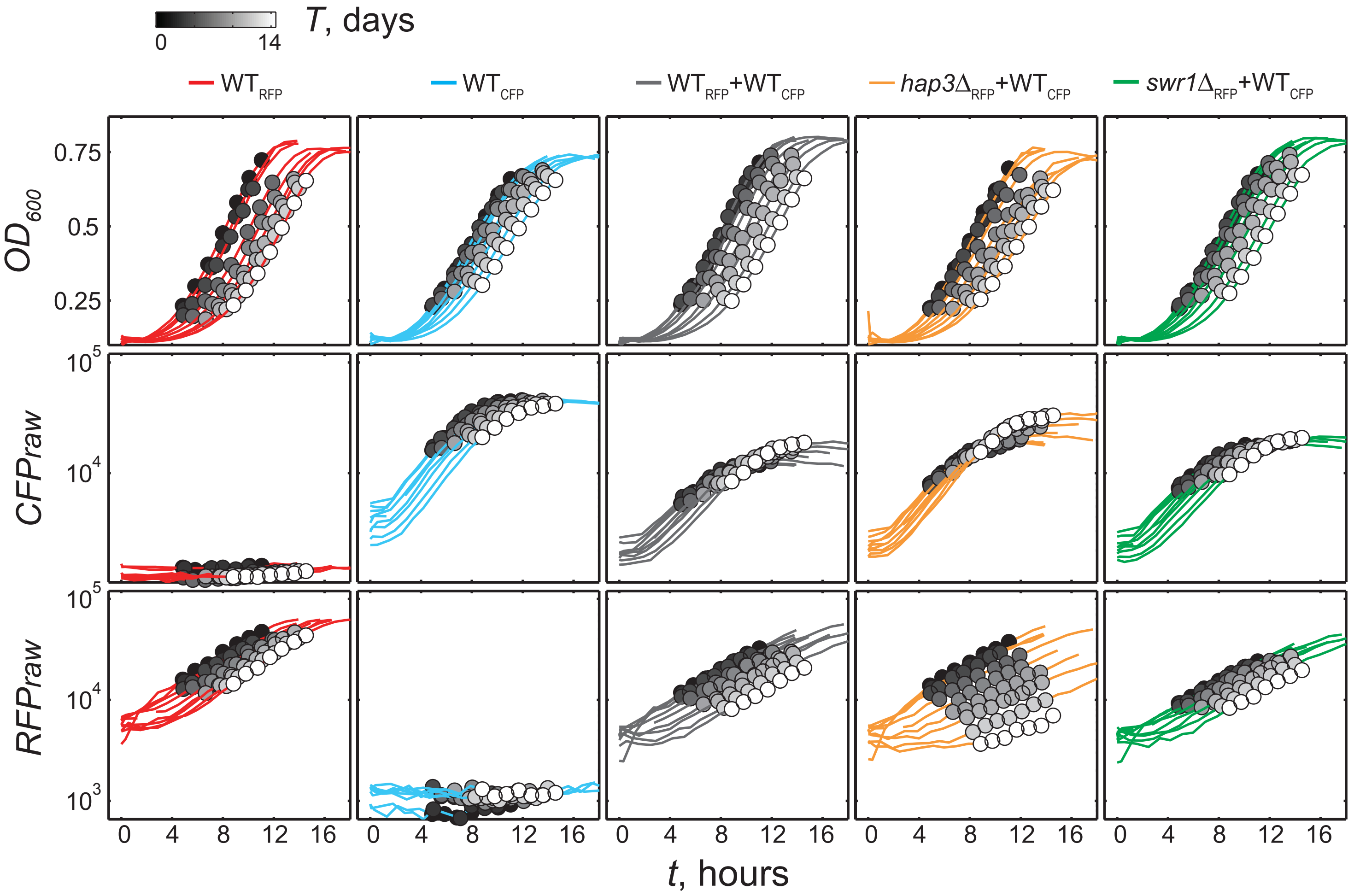

Supplementary Figure S1. Examples of raw data for OD_{600}, and *RFPraw* and *CFPraw* signal from outgrowth-culture kinetics monitored throughout the experiment**.** Each data-series curve corresponds to one outgrowth at time T (0-14 days) in stationary phase. Selected experiments are shown in five columns, from left to right: Only WT_{RFP} (red lines), only WT_{CFP} (blue lines), both WT populations in co-culture (WT_{RFP}+WT_{CFP}, gray lines), a short-lived deletion strain in co-culture with the WT (*hap3*Δ_{RFP}+WT_{CFP}, orange lines), and a long-lived strain with the WT (*swr1*Δ_{RFP} +WT_{CFP}, green lines). Samples are measured throughout the outgrowth culture after inoculation, but data analysis only considers data points at exponential growth (circles in grayscale).
