# Supplementary material for: An Optimized Competitive-Aging Method Reveals Gene-Drug Interactions Underlying the Chronological Lifespan of Saccharomyces cerevisiae
Source: Front Genet. 2020 May 14;11:468. doi: 10.3389/fgene.2020.00468 (PMC7240105; doi:10.3389/fgene.2020.00468)
Supplement: FIGURE S1 — Examples of raw data for OD600, and RFPraw and CFPraw signal from outgrowth-culture kinetics monitored throughout the experiment. [file Data_Sheet_1.zip › 04-AVELAR_FigS2.pdf]

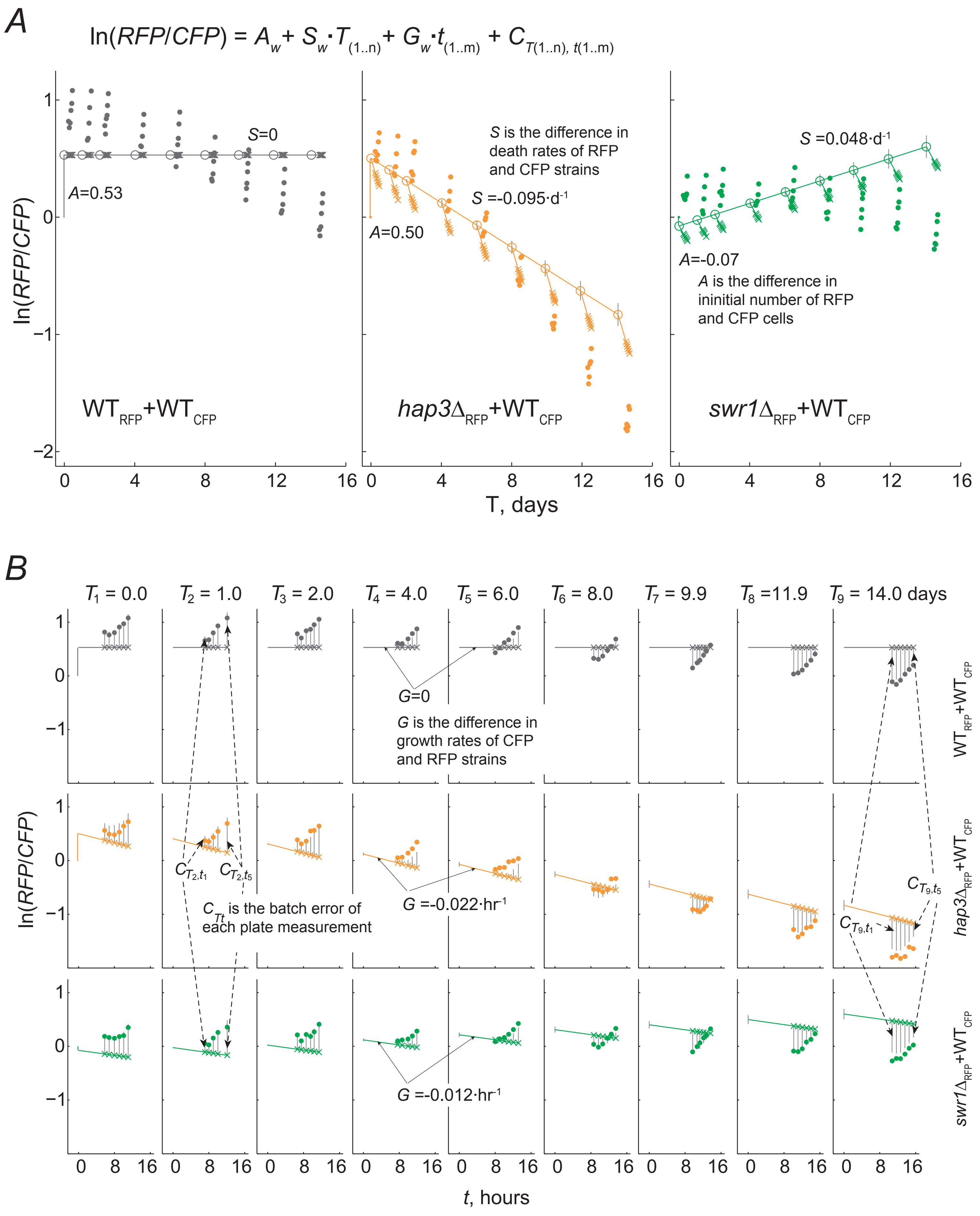
A
ln(RFP/CFP) = A_w + S_w·T_(1..n) + G_w·t_(1..m) + C_T(1..n), t(1..m)
ln(RFP/CFP)
S=0
A=0.53
WT_RFP+WT_CFP
S is the difference in death rates of RFP and CFP strains
S =-0.095·d⁻¹
A=0.50
hap3Δ_RFP+WT_CFP
S =0.048·d⁻¹
A=-0.07
A is the difference in ininitial number of RFP and CFP cells
swr1Δ_RFP+WT_CFP
T, days
B
T1 = 0.0
T2 = 1.0
T3 = 2.0
T4 = 4.0
T5 = 6.0
T6 = 8.0
T7 = 9.9
T8 =11.9
T9 = 14.0 days
G=0
G is the difference in growth rates of CFP and RFP strains
C_T2,t1
C_T2,t5
C_Tt is the batch error of each plate measurement
G =-0.022·hr⁻¹
C_T9,t5
C_T9,t1
G =-0.012·hr⁻¹
WT_RFP+WT_CFP
hap3Δ_RFP+WT_CFP
swr1Δ_RFP+WT_CFP
t, hours

(...)

Supplementary Figure S2. Multiple linear modeling fits relative survivorship and relative growth rate from competitive-aging cultures. Examples of a reference competition (gray) and two deletion strains in co-culture with the WT_{CFP} (*hap3*$\Delta_{RFP,}$ orange; *swr1*Δ_{RFP}, green). In both panels, dots indicate the experimental ln(*RFP/CFP*) measurements. ***A,*** Fitted data is shown in the T timescale (days). Open circles are the data fitted at T_i and error bars at those time points are the 95% CI of the fit; their linear regression has slope S (the relative survivorship, our parameter of interest). For each well, parameter A indicates the deviation from ln(*RFP/CFP*)=0 at T=0, as indicated by the vertical line at T=0. ***B,*** Fitted data is shown in the t timescale (hours); nine panels correspond to outgrowths at different age times, *T*. Crosses shown directly below or above experimental data are the fitted data $ln(\frac{RFP}{CFP})_{T_i,\, t_j}$ at the time of each measurement; their linear regression has slope G (the relative growth rate). All samples (wells) in the same 96-well plate measurement have the same $C_{T,t}$ (systematic batch error, gray vertical lines).
