# Supplementary material for: An Optimized Competitive-Aging Method Reveals Gene-Drug Interactions Underlying the Chronological Lifespan of Saccharomyces cerevisiae
Source: Front Genet. 2020 May 14;11:468. doi: 10.3389/fgene.2020.00468 (PMC7240105; doi:10.3389/fgene.2020.00468)
Supplement: FIGURE S1 — Examples of raw data for OD600, and RFPraw and CFPraw signal from outgrowth-culture kinetics monitored throughout the experiment. [file Data_Sheet_1.zip › 05-AVELAR_FigS3.pdf]

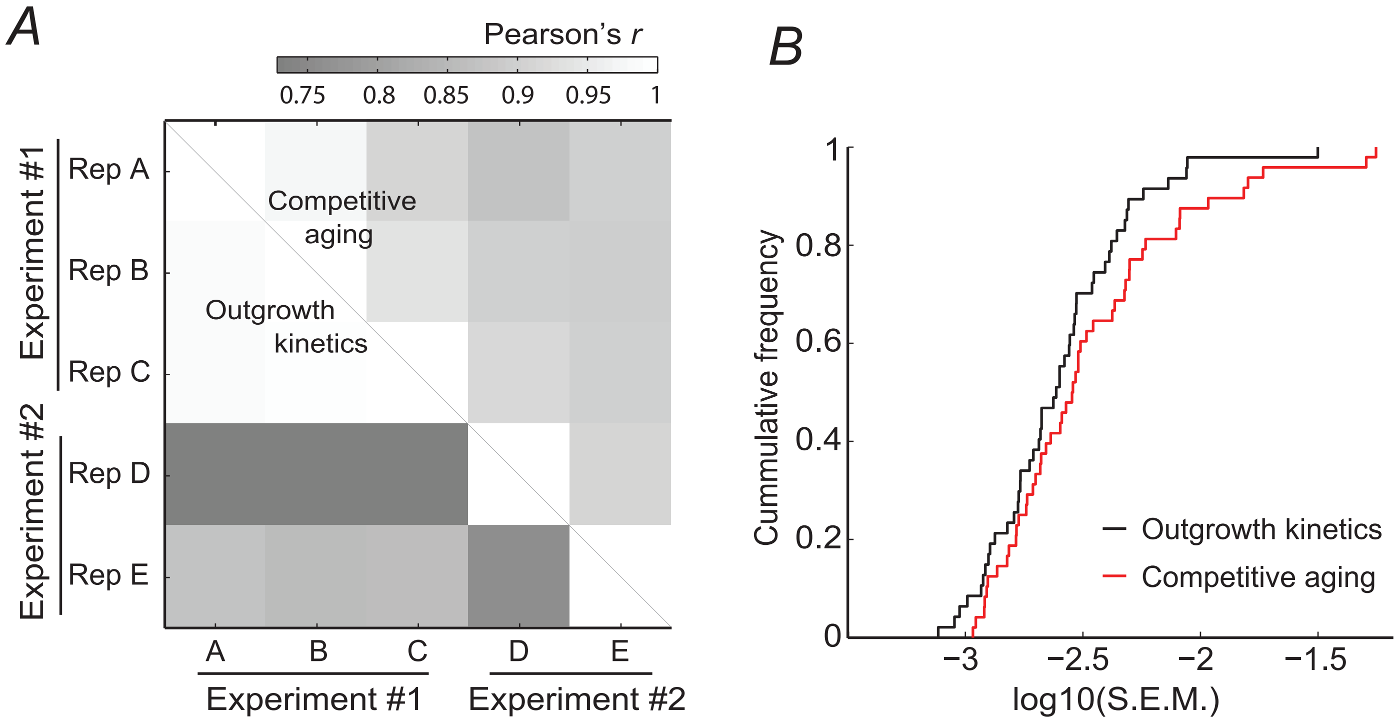

Supplementary Figure S3. Comparing replicability of CLS measurements from two methods. ***A*****,** Paired Pearson's *r* correlation coefficient among the relative survivorship, *S*, from three (experiment #1, plates A, B, and C) or two replicates (experiment #2, plates C and D). Competitive-aging correlations are shown above, while OD-kinetics in monoculture are shown below the diagonal. ***B*****,** Cumulative S.E.M. (intra-experiment) of all mutant´s *S*. Data of the five experimental batches are shown in each series (n=47 and n=48 for monoculture outgrowth kinetics and competitive aging, respectively). The median S.E.M. of *S* from both methods are statistically indistinguishable (p=0.154, Wilcoxon rank sum test).
