# Supplementary material for: An Optimized Competitive-Aging Method Reveals Gene-Drug Interactions Underlying the Chronological Lifespan of Saccharomyces cerevisiae
Source: Front Genet. 2020 May 14;11:468. doi: 10.3389/fgene.2020.00468 (PMC7240105; doi:10.3389/fgene.2020.00468)
Supplement: FIGURE S1 — Examples of raw data for OD600, and RFPraw and CFPraw signal from outgrowth-culture kinetics monitored throughout the experiment. [file Data_Sheet_1.zip › 06-AVELAR_FigS4.pdf]

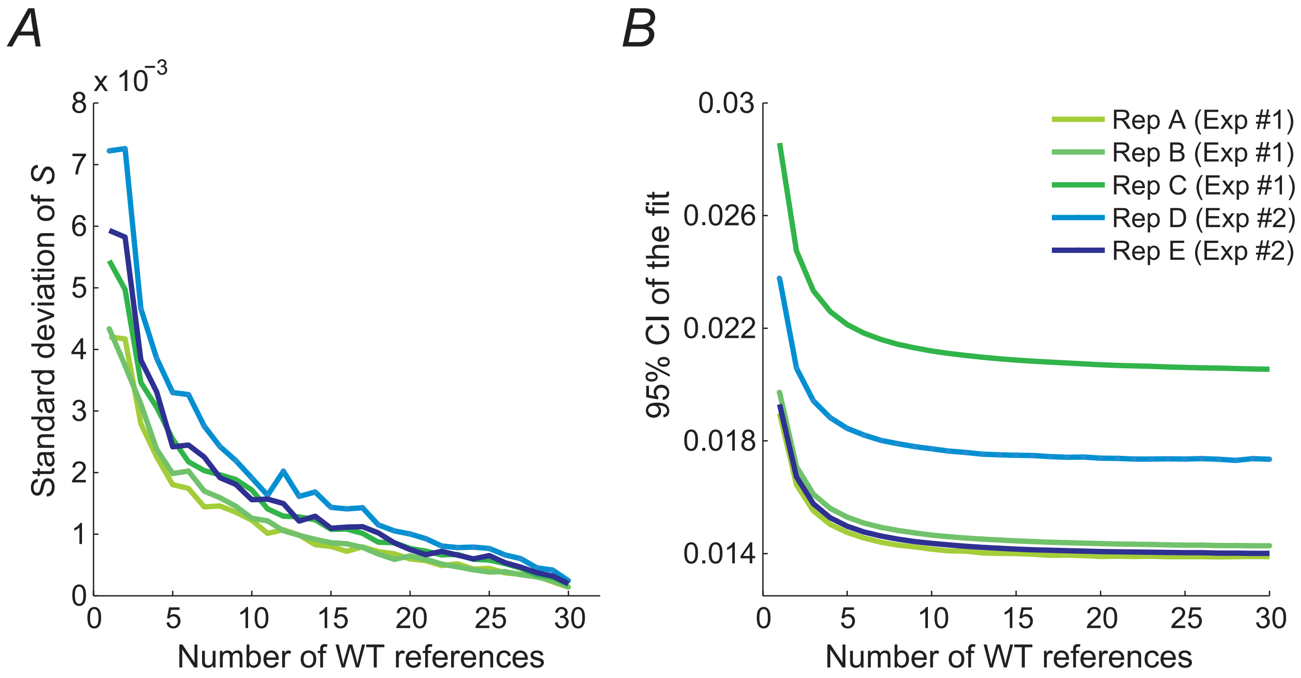

Supplementary Figure S4. Variation in S decreases with the number of reference samples used. Thirty-one WT_{RFP}+WT_{CFP} reference competitions were used in each plate. To estimate the effects of increasing number of reference samples, the model was fed with different numbers of reference wells and the reminder control samples were treated as additional mutant samples. For each given number of reference samples, 100 permutations were randomly selected and the selection coefficient (S) of all mutant strains was calculated accordingly. ***A***, The variation using different permutations was quantified as the standard deviation of each mutant sample; the average standard deviation of S of all mutant samples is shown as a function of the number of reference samples in the model. ***B*,** The fit to the model was calculated along with the 95% CIs; the difference between the upper and lower CI bounds is plotted against the number of reference samples added to the model.
