# Supplementary material for: An Optimized Competitive-Aging Method Reveals Gene-Drug Interactions Underlying the Chronological Lifespan of Saccharomyces cerevisiae
Source: Front Genet. 2020 May 14;11:468. doi: 10.3389/fgene.2020.00468 (PMC7240105; doi:10.3389/fgene.2020.00468)
Supplement: FIGURE S1 — Examples of raw data for OD600, and RFPraw and CFPraw signal from outgrowth-culture kinetics monitored throughout the experiment. [file Data_Sheet_1.zip › 07-AVELAR_FigS5.pdf]

Figure S5

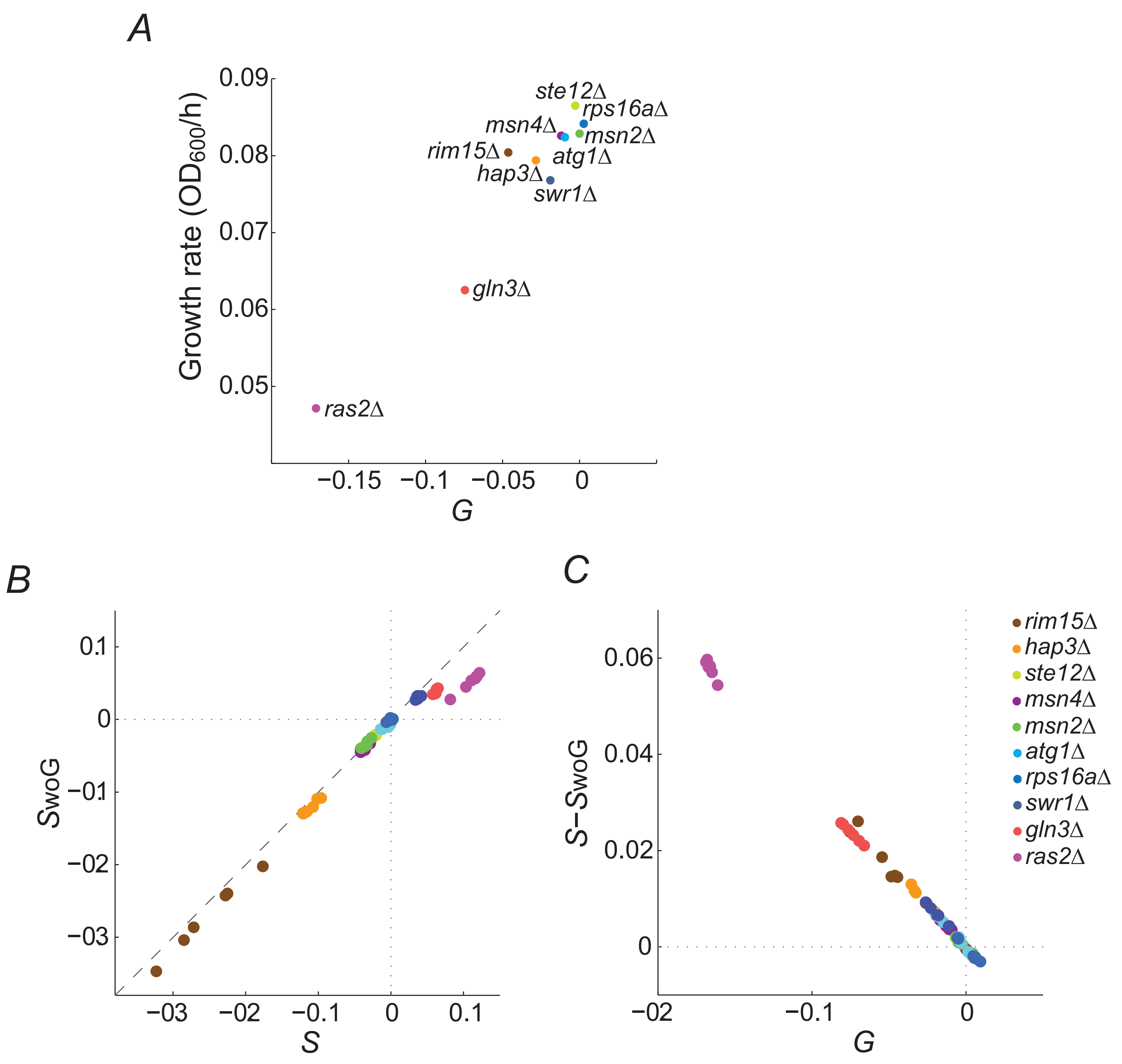

Supplementary Figure S5. The parameter G corrects effects of differential growth rates that otherwise affect relative survivorship, S. ***A***, The actual growth rate at exponential phase of gene-deletion strains measured as average increase in OD_{600} with time (*n*=6-7), compared to the modeled parameter G. ***B***, Scatter plot showing the average S value from 3-5 replicates with parameter G is included in the multiple linear regression (horizontal axis) and when the regression is run without G (S_{woG}, vertical axis). ***C,*** The mild difference between calculations of S showed in panel B is mostly explained by changes in the parameter G.
