# Supplementary material for: An Optimized Competitive-Aging Method Reveals Gene-Drug Interactions Underlying the Chronological Lifespan of Saccharomyces cerevisiae
Source: Front Genet. 2020 May 14;11:468. doi: 10.3389/fgene.2020.00468 (PMC7240105; doi:10.3389/fgene.2020.00468)
Supplement: FIGURE S1 — Examples of raw data for OD600, and RFPraw and CFPraw signal from outgrowth-culture kinetics monitored throughout the experiment. [file Data_Sheet_1.zip › 08-AVELAR_FigS6.pdf]

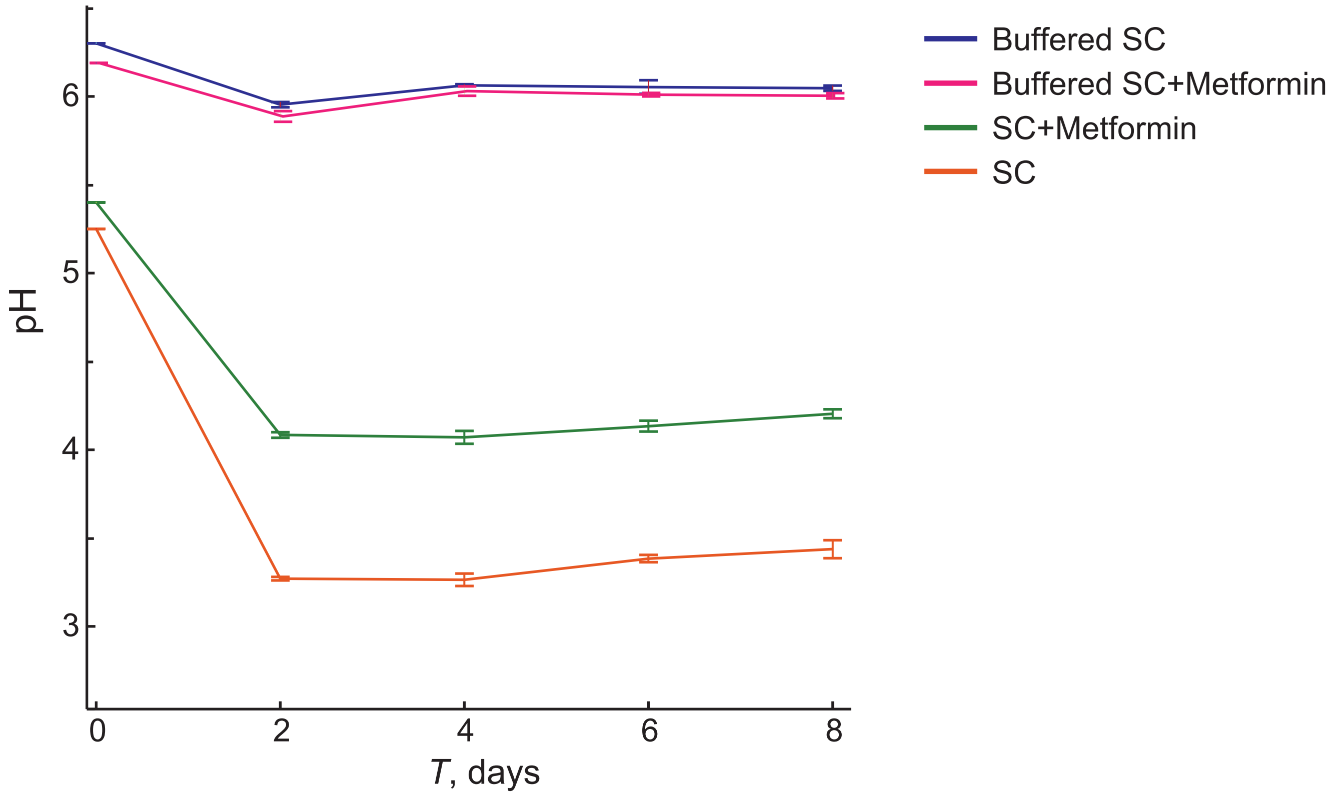

Supplementary Figure S6. Metformin limits media acidification in stationary phase. Plot shows the measured pH of aging cultures as a function of time in stationary phase for SC medium (orange), SC with metformin (green) with buffered-SC medium (blue), and buffered-SC medium with metformin (magenta). Medium was buffered as previously reported (Burtner et al. 2006). The mean of three replicates with standard deviation is shown. Data was collected by pooling media from three wells of the semi-deep well plate (~2.0 mL total volume) and measured in a pH meter (Oakton Ion-510) at the indicated time points (days) after stationary phase.
