# Supplementary material for: An Optimized Competitive-Aging Method Reveals Gene-Drug Interactions Underlying the Chronological Lifespan of Saccharomyces cerevisiae
Source: Front Genet. 2020 May 14;11:468. doi: 10.3389/fgene.2020.00468 (PMC7240105; doi:10.3389/fgene.2020.00468)
Supplement: FIGURE S1 — Examples of raw data for OD600, and RFPraw and CFPraw signal from outgrowth-culture kinetics monitored throughout the experiment. [file Data_Sheet_1.zip › 09-AVELAR_FigS7.pdf]

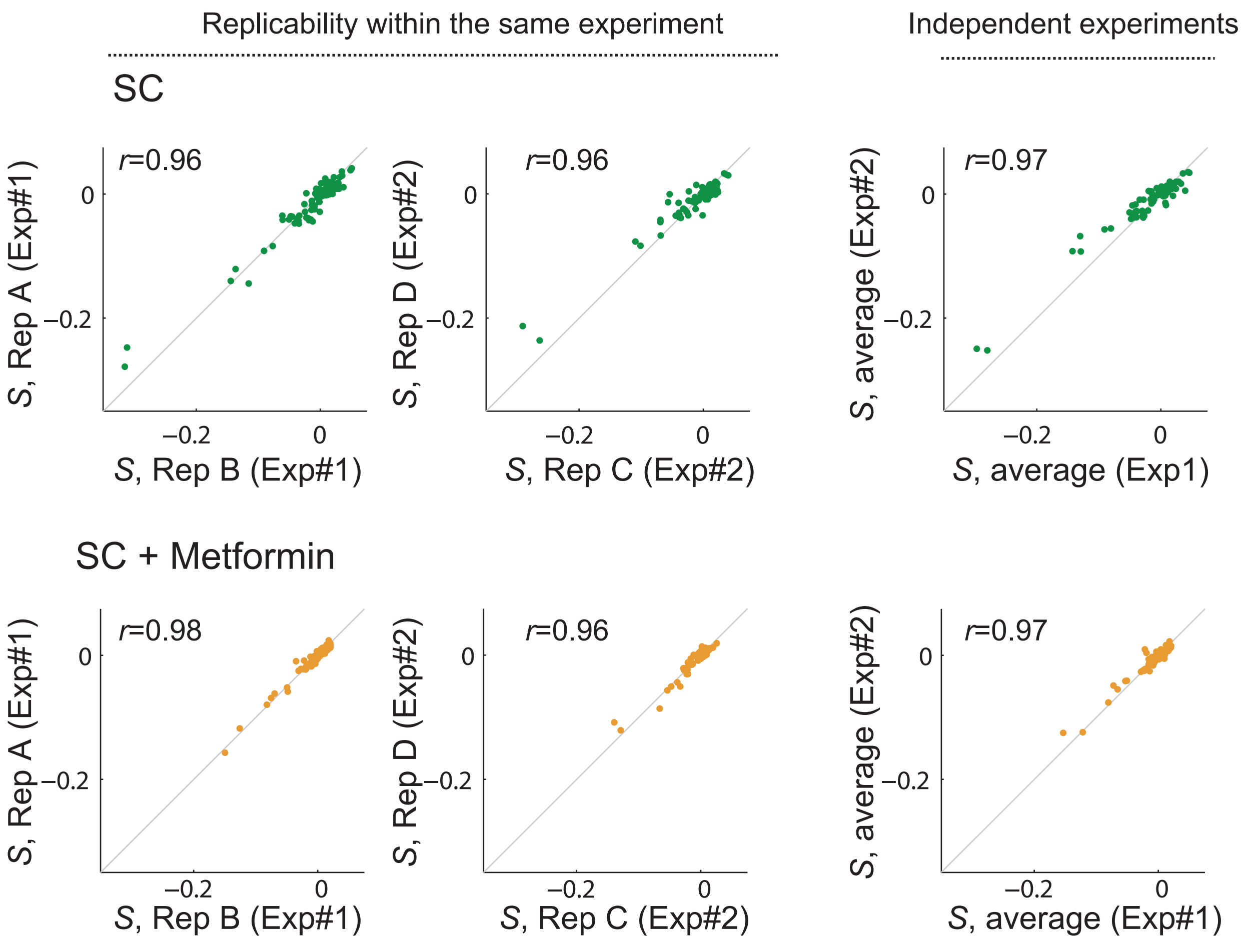

Supplementary Figure S7. The relative survivorship, S, is well replicated within the same experiment (replicates 'A' and 'B', or 'C' and 'D') and between different experiments (#1 and #2), both without (green) and with metformin (orange). Left and central panels show single data points comparing two replicate plates, while the right panel shows the comparison of the averaged data of two replicates within each independent experiment. The Pearson correlation coefficients are shown in each panel.
