# Supplementary material for: An Optimized Competitive-Aging Method Reveals Gene-Drug Interactions Underlying the Chronological Lifespan of Saccharomyces cerevisiae
Source: Front Genet. 2020 May 14;11:468. doi: 10.3389/fgene.2020.00468 (PMC7240105; doi:10.3389/fgene.2020.00468)
Supplement: FIGURE S1 — Examples of raw data for OD600, and RFPraw and CFPraw signal from outgrowth-culture kinetics monitored throughout the experiment. [file Data_Sheet_1.zip › 10-AVELAR_FigS8.pdf]

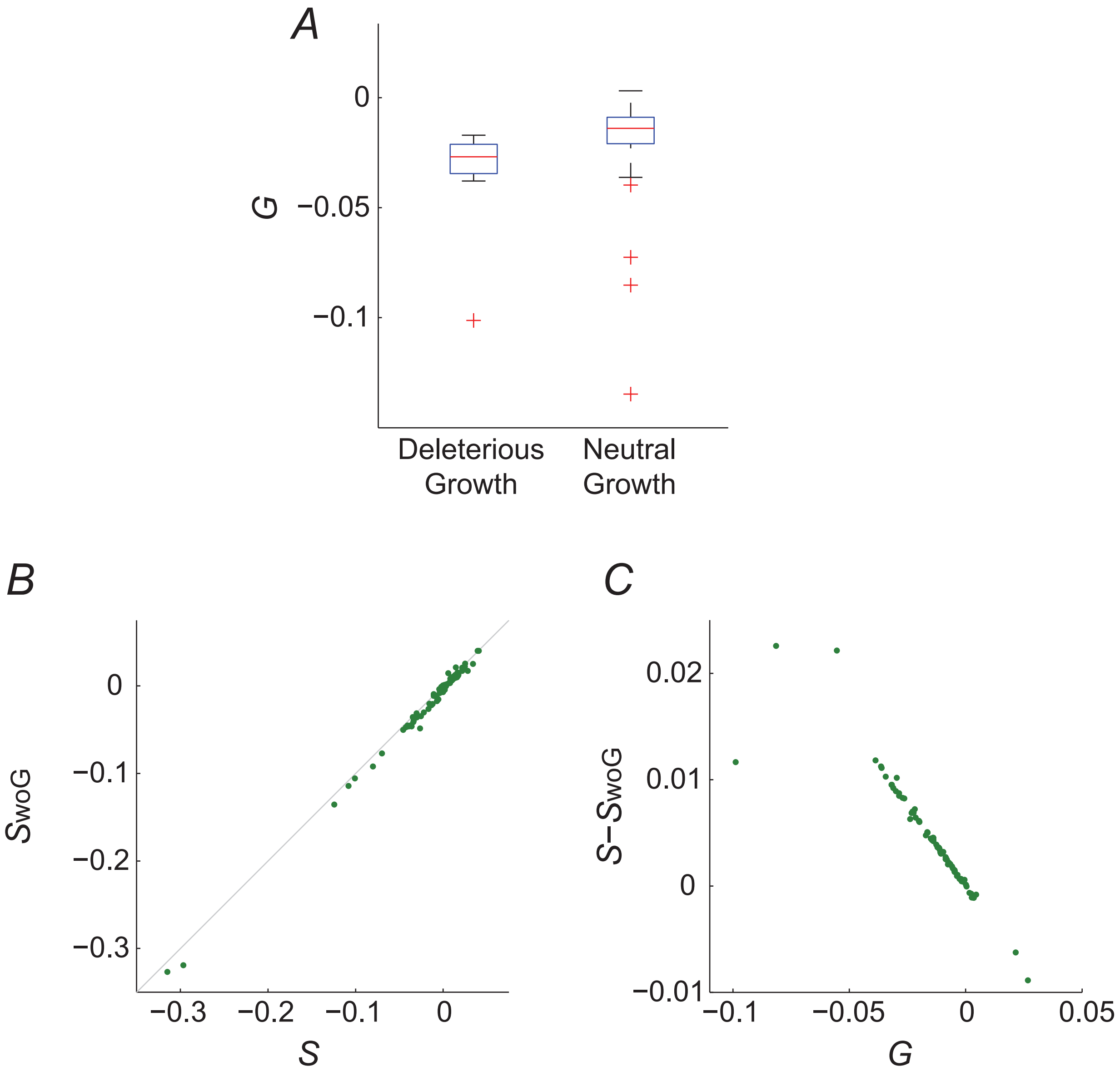

Supplementary Figure S8. The parameter G corrects effects of differential growth rates that otherwise affect relative survivorship, S. ***A***, Box plots of the G calculated for mutants with reduced fitness or without effects as previously reported (Costanzo et al. 2010). Deleterious knockouts, (fitness f<0.95, n=10) usually have a more negative G value than neutral knockouts without known growth defects (f≥0.95, n=67) (p=0.0014, Wilcoxon rank sum test). ***B***, Scatter plot showing the obtained S value with the parameter G is included in the multiple linear regression (horizontal axis) and when the regression runs without G (S_{woG}, vertical axis). ***C*,** The difference between calculations of S showed in panel A is mostly explained by average changes in the parameter G.
