# Supplementary material for: An Optimized Competitive-Aging Method Reveals Gene-Drug Interactions Underlying the Chronological Lifespan of Saccharomyces cerevisiae
Source: Front Genet. 2020 May 14;11:468. doi: 10.3389/fgene.2020.00468 (PMC7240105; doi:10.3389/fgene.2020.00468)
Supplement: FIGURE S1 — Examples of raw data for OD600, and RFPraw and CFPraw signal from outgrowth-culture kinetics monitored throughout the experiment. [file Data_Sheet_1.zip › 11-AVELAR_FigS9.pdf]

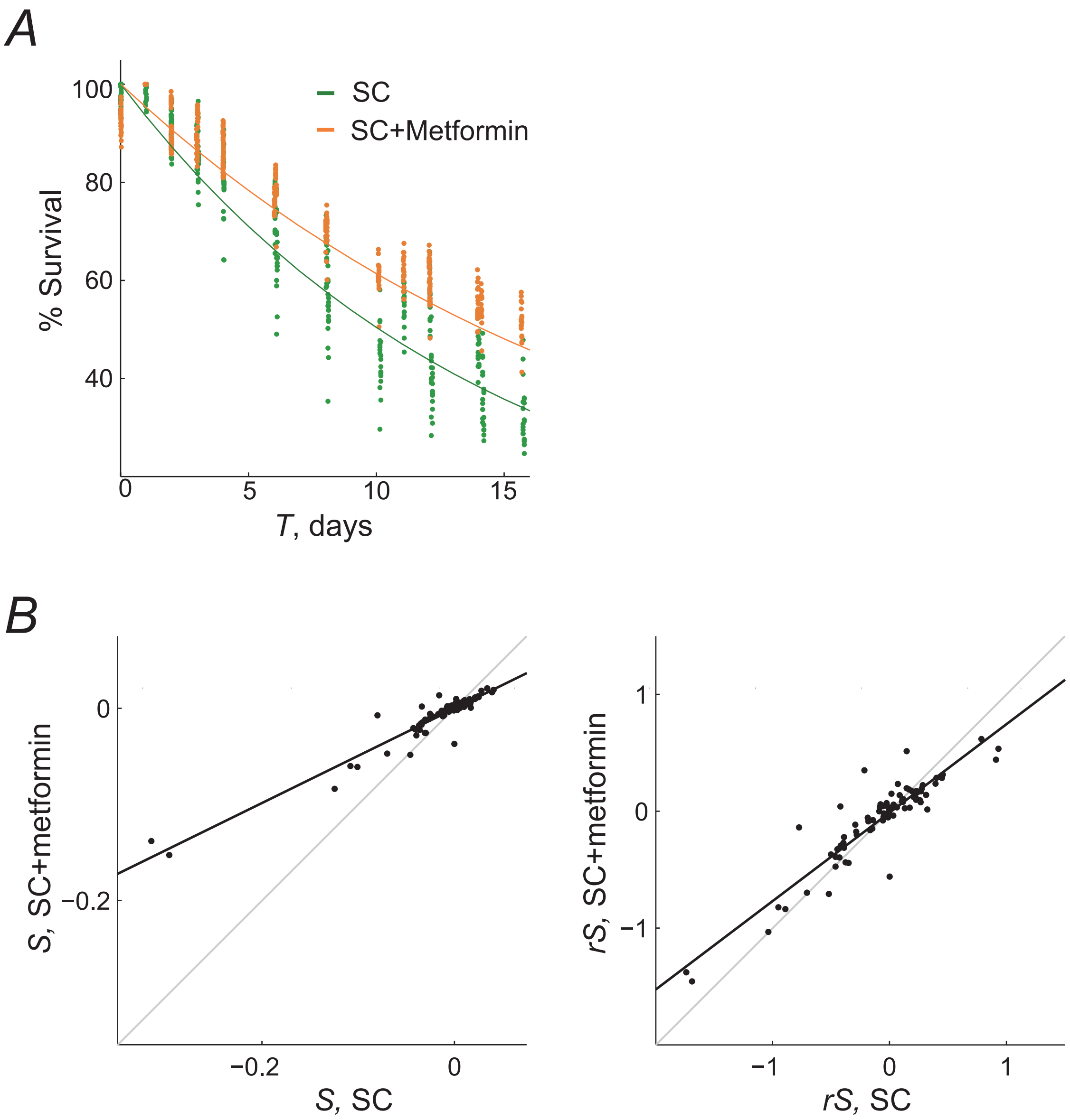

Supplementary Figure S9. Relative survivorship (r*S*) data rescaling. To quantitatively compare the relative phenotypes between two conditions of different WT phenotype, data were rescaled to a dimensionless relative survival parameter, $r\,S$. ***A***, The death rates of the WT without (green) and with metformin (orange) were calculated by fitting to an exponential function the percent survival at different days of aging. Data points were from ten replicate samples in four replicate experiments (*n*=39 with metformin and *n*=40 for SC); lines are the average fits. ***B***, The relative survival S shows a bias between the two conditions (left); the rescaled survivorship parameter $r\,S$ provides a better comparison under two conditions in which the WT dies at different rates (right). Specifically, relative survivorship was rescaled with the formula $r\,S = -\log\left(1 - \frac{S}{r_{w\,t}}\right)$ using the average wild-type death rate obtained in each plate.
