# Supplementary material for: An Optimized Competitive-Aging Method Reveals Gene-Drug Interactions Underlying the Chronological Lifespan of Saccharomyces cerevisiae
Source: Front Genet. 2020 May 14;11:468. doi: 10.3389/fgene.2020.00468 (PMC7240105; doi:10.3389/fgene.2020.00468)
Supplement: FIGURE S1 — Examples of raw data for OD600, and RFPraw and CFPraw signal from outgrowth-culture kinetics monitored throughout the experiment. [file Data_Sheet_1.zip › 12-AVELAR_FigS10.pdf]

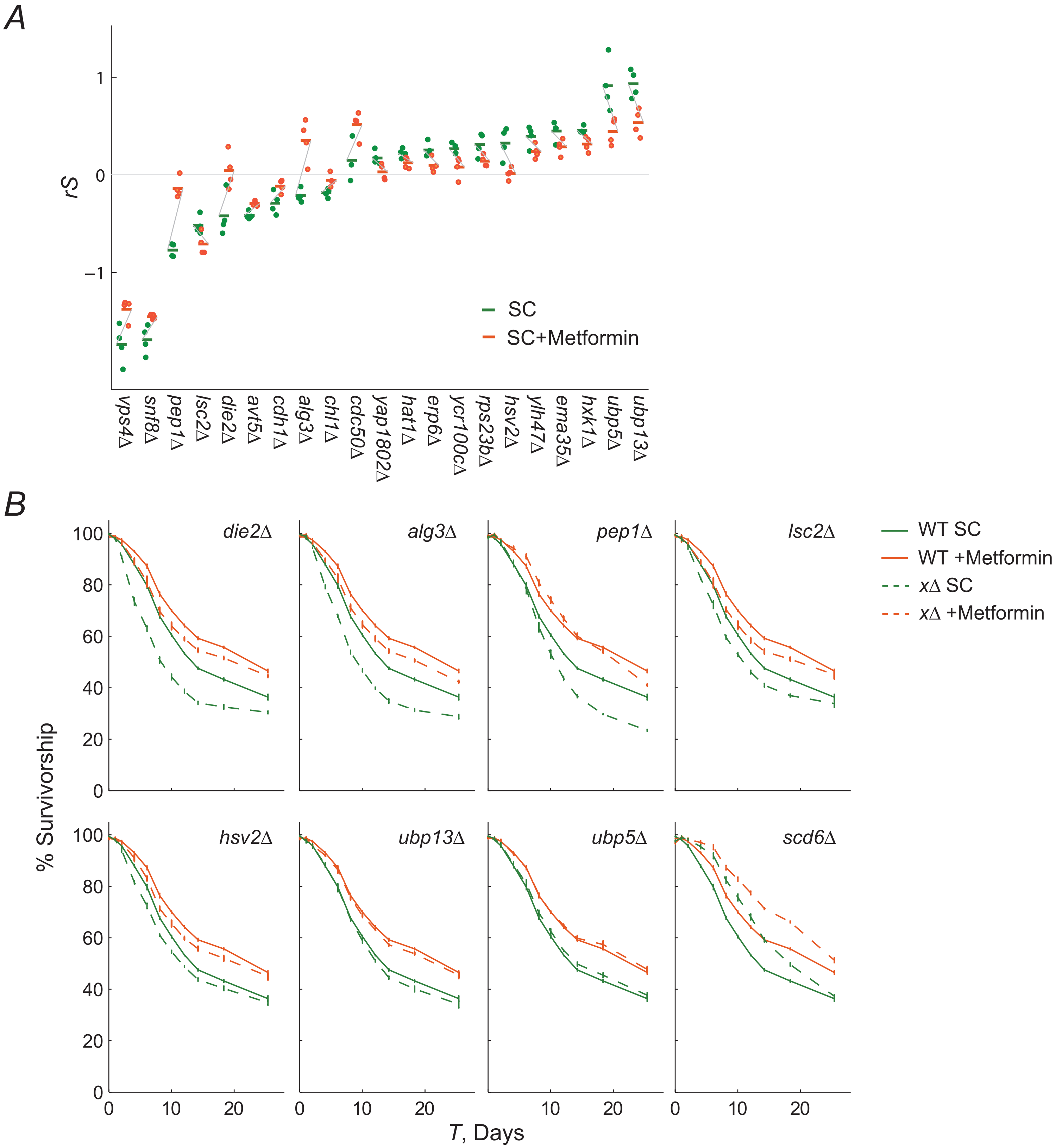

Supplementary Figure S10. Candidate gene-drug interactions and validation. ***A,*** Plot shows individual and average measurements of rS of strains with differential phenotypic effects (p<0.05, t-test). Gray diagonal lines are used to visualize the sign of the gene-drug interaction. ***B,*** Validation of gene-drug interactions using the OD kinetics method. Survival curves were determined through aging monocultures of eight gene-deletion strains with some of the most extreme short- or long-lived phenotypes in SC, including candidate gene-drug interactions. Average percentage of survivorship is shown for the wild-type (solid lines) and gene-deletion strains (dashed lines) in both conditions, SC (green) and SC+Metformin (orange); error bars indicate the S.E.M (n=4).
